# Exploring Cyclodextrin-Based Nanosponges as Drug Delivery Systems: Evaluation of Spectroscopic Methods for Examining Structure and Dynamics of Nanosponges

**DOI:** 10.3390/ijms26199342

**Published:** 2025-09-24

**Authors:** Bartłomiej Pyrak, Karolina Rogacka-Pyrak, Tomasz Gubica

**Affiliations:** 1 Doctoral School, Medical University of Warsaw, Żwirki i Wigury 81, 02-093 Warsaw, Poland; bartlomiej.pyrak@wum.edu.pl; 2 Department of Organic and Physical Chemistry, Faculty of Pharmacy, Medical University of Warsaw, Banacha 1, 02-097 Warsaw, Poland; 3 First Department of Psychiatry, Institute of Psychiatry and Neurology, Sobieskiego 9, 02-957 Warsaw, Poland; karolinarogacka2697@gmail.com

**Keywords:** cyclodextrin, nanosponge, spectroscopic methods, structure–property relationship

## Abstract

Cyclodextrin-based nanosponges (CDNSs) are novel polymers composed of cross-linked cyclodextrin (CD) macrocyclic units, whose characteristics make them great candidates for drug delivery systems with adjustable properties for the drug release process. Examination of the molecular structure and dynamics of CDNSs is a necessary starting point in the first step toward their broad application. Spectroscopic methods are effective analytical tools for probing the structure–property relationships of polymer structures. Infrared (IR) and Raman spectroscopies provide insight into the behavior of hydrogen bond (H-bond) networks influencing the properties of CDNS polymeric networks. Scattering techniques such as inelastic neutron scattering (INS) and Brillouin light scattering (BLS) probe elastic properties, while small-angle neutron scattering (SANS) examines the structural inhomogeneities and water sorption abilities of CDNS materials. Complete evaluation is possible using nuclear magnetic resonance (NMR), which can provide data on CDNS network dynamics. This article summarizes the results of a wide examination of CDNSs with the use of spectroscopic methods and reveals the links between the microscopic behavior and macroscopic properties of CDNSs, enabling the customization of their properties for various biomedical purposes.

## 1. Introduction

**Figure 1:**
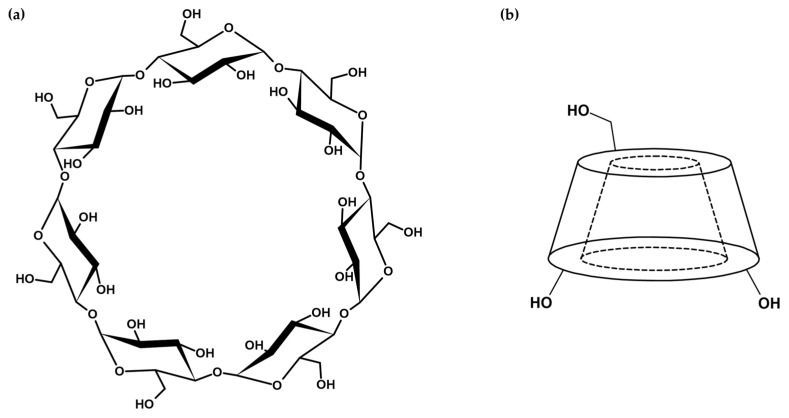
(**a**) Chemical structure of β-CD composed of seven glucose units linked via α-1,4-glycosidic bonds. (**b**) Schematic representation of β-CD structure (hollow truncated cone) with labeled primary and secondary OH groups at narrow and wide rims, respectively.

**Figure 2:**
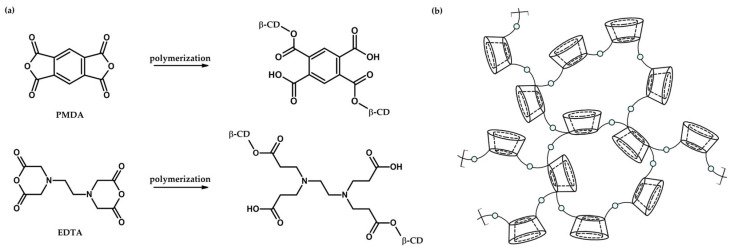
(**a**) Evolution of ester cross-linkers in the polymerization process. During the ring-opening process, four active carbonyl moieties are formed; two of them, situated at opposite ends of the cross-linker molecule, interact with the CD unit and create an ester bond, while the remaining two become free carboxyl groups. (**b**) Schematic representation of CDNS structure (blue dots represent cross-linker molecules, which bind predominantly to primary OH groups of CD units [10]).

Cyclodextrin-based nanosponges (CDNSs) are polymeric scaffolds composed of cyclodextrin (CD) units linked via molecules working as cross-linking agents. The properties of CDNSs are tunable at the synthesis level through the choice of cyclodextrin and cross-linker type and their relative molar ratio 1:*n*, with *n* being the number of cross-linker molecules per CD unit. Due to its thoroughly studied structure and properties and relatively low production cost, β-cyclodextrin (β-CD, Figure 1) became the go-to molecule for the synthesis of CDNSs [1,2,3]; however, α-CD, γ-CD and derivatives of β-CD are also universally used due to their specific physicochemical features [4]. Depending on the cross-linker type, CDNSs can be divided into carbamate CDNSs (cross-linked using hexamethylene diisocyanate (HMDI) or toluene-2,4-diisocyanate (TDI)), carbonate CDNSs (cross-linked using diphenyl carbonate (DPC) or 1,1′-carbonyldiimidazole (CDI)) and ester CDNSs (cross-linked using pyromellitic dianhydride (PMDA) or ethylenediaminetetraacetic dianhydride (EDTA), Figure 2a). The last group is of significant importance due to the water absorption properties of these CDNSs, resulting in the formation of hydrogels, which are generally used as novel drug delivery systems [5,6,7,8,9]. Examination of the molecular structure of CDNSs is difficult, as their polymeric nature arises from random polymerization which, despite knowing its mechanism and structural preferences, is still not fully understood (Figure 2a,b) [10]. Gelation of CDNSs or drug incorporation results in additional changes in CDNS behavior, which further complicates this already difficult conjuncture. Nonetheless, investigation of CDNS structure-related properties is possible by studying changes in structure and dynamics at the molecular level resulting from alterations in macroscopic parameters of the system, such as temperature, hydration level and pH, to which CDNS responses vary. This work summarizes the knowledge on fluctuations in PMDA- and EDTA-based CDNS properties resulting from the abovementioned alterations observed using spectroscopic methods, including infrared (IR) and Raman spectroscopies, inelastic neutron scattering (INS), Brillouin light scattering (BLS), small-angle neutron scattering (SANS) and nuclear magnetic resonance (NMR) techniques. The main objective of this text is to demonstrate the precision and reliability of spectroscopic methods in examining CDNS networks and to point out the links between the microscopic and macroscopic properties of CDNSs, information that may be used as a gateway for the tunability of their drug release properties [11]. Table 1 summarizes original articles regarding the use of spectroscopic methods to examine the structure–property relationships of ester nanosponges. More in-depth information about the synthesis and physicochemical properties of β-CD and CDNS is reported elsewhere [11,12,13,14,15,16,17,18,19,20,21,22,23,24].

## 2. IR and Raman Spectroscopies

**Figure 3:**
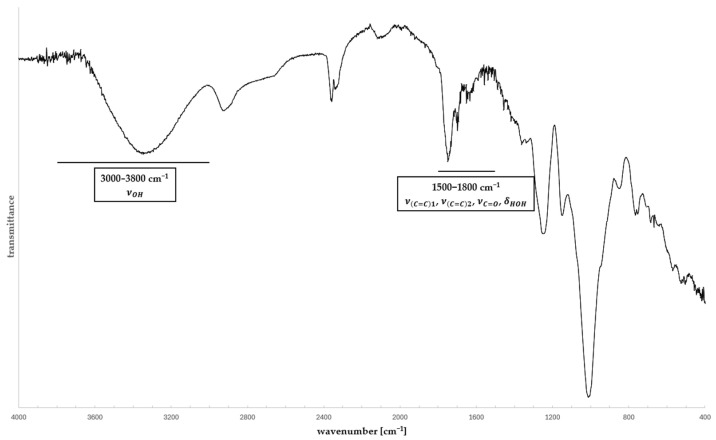
ATR-FT-IR spectrum of CDNS. Spectral regions with characteristic vibrational modes undergoing evaluation are marked on the spectrum with signals.

Despite obvious similarities, the activity of vibrational modes differs between IR and Raman spectroscopies based on their individual selection rules [51,52]. Excitation of vibrational or rotational modes resulting in changes in dipole moments enables interaction with electromagnetic radiation and makes the mode IR-active. This feature makes IR sensitive to heteronuclear groups and polar bonds; thus, it is the go-to method for analyzing functional groups. Raman-active modes are observed as a result of changes in polarizability, representing the distortion of an electron cloud in response to the external electromagnetic field, making Raman spectroscopy more sensitive to homonuclear bonds and suitable for analyzing complex organic structures. Fourier-transformed IR (FT-IR) and Raman spectroscopies are effective methods for investigating molecular structures because the position and shape of the peaks are highly sensitive to environmental changes and intermolecular interactions. Due to their distinct selection rules, these methods are complementary in the examination of the structures of organic molecules [53]. CDNS structure is evaluated in two spectral regions—3000–3800 cm^−1^, dominated by O-H bond stretching $\nu_{OH}$, and 1500–1800 cm^−1^, rich in the cross-linker-related modes $\nu_{\left(C=C\right)1}$ (ring breathing mode of PMDA aromatic ring), $\nu_{\left(C=C\right)2}$ (aromatic ring stretching with C-H bond bending) and $\nu_{C=O}$ (carbonyl groups of cross-linkers), as well as the HOH bending mode $\delta_{HOH}$ of water molecules [43] (Figure 3).

### 2.1. $\nu_{OH}$—OH Stretching Band

#### 2.1.1. General Analysis

Due to the sub-picosecond lifetime of H-bonds [54], the structure of the hydrogen bond (H-bond) network is constantly reorganized and can be studied through the observation of $\nu_{OH}$ modes. Changes in the shape or position of $\nu_{OH}$ are related to changes in the strength, intermolecular distances and cooperativity of a H-bond network created by water molecules confined in nanochannels or attached to the CDNS surface [32,55]. An upshift of $\nu_{OH}$ is related to a decrease in H-bond lifetime and the destruction of the H-bond network, while a downshift is associated with the strengthening of H-bonds and reorganization of the H-bond network into highly coordinated structures.

The $\nu_{OH}$ position varies with 1:*n*—the initial upshift with increasing 1:*n* is related to the destructive effect of increasing reticulation on H-bond networks. The maximum upshift obtained for a 6-fold excess of cross-linker is connected to a perfect balance between the OH groups of CD and the activated carbonyl groups of PMDA/EDTA, leading to the formation of the densest polymeric network, which disrupts H-bond networks to the highest degree. A further increase in 1:*n* is reflected by a downshift of $\nu_{OH}$ due to the preference for water–water over water–polymer interactions, resulting in the development of coordinated H-bond networks [28].

The H-bond networks of hydrogels are strongly affected by changes in hydration level h (ratio between mass of water used in hydration of CDNS and mass of swollen polymer), which results in the redistribution of water molecules. An increase in h results in an initial upshift of $\nu_{OH}$, which can be explained by the interaction of water molecules with the CDNS surface, leading to the formation of a less coordinated H-bond network relative to highly coordinated networks. A further increase in h results in a $\nu_{OH}$ downshift due to saturation of water binding sites at the CDNS surface, allowing the formation of a highly interconnected network between “surplus” water molecules [28,32].

Registration of FT-IR spectra at different temperatures enables exploration of changes in H-bond network properties related to thermal disruption. Vibrational data of CDNS hydrogels show $\nu_{OH}$ upshifts corresponding to the destructive effect of thermal motion on H-bond structure [28,40,43].

#### 2.1.2. Sub-Band Analysis

Apart from generalized H-bond changes, the broad $\nu_{OH}$ band also contains information about different populations of O-H bonds that arise from different dynamical responses of local H-bond networks to external stimuli [28]. Band decomposition procedures can be used to extract specific information about different types of H-bonds by identifying overlapping contributions (sub-bands) within a broad band. The second derivative of the spectrum reveals distinct minima representing the positions of sub-bands’ maxima. Band decomposition of $\nu_{OH}$ of dry CDNS reveals five sub-bands: $\omega_{1}$ (3100–3150 cm^−1^) and $\omega_{3}$ (3300–3350 cm^−1^) of interstitial water molecules (related to H-bonds between water molecules and between water and CD unit surface); $\omega_{2}$ (3200–3300 cm^−1^) of secondary OH groups of CD units; $\omega_{4}$ (3350–3450 cm^−1^) of primary OH groups of CD units; and $\omega_{5}$ (3500–3550 cm^−1^) of intracavity water molecules [27]. Based on the position of sub-bands’ maxima, fitting procedures are implemented to resolve the shape of each contribution, most commonly by using Voigt functions (Appendix A.1) [55]. By calculating the area under the curve, the percentage areas of sub-bands related to population sizes of H-bonded molecules ($I_{1}-I_{5}$) can be obtained [27]. With increasing 1:*n*, two changes are observed: $I_{2}$ increases as a result of enhanced steric hindrances, which disrupt the molecular symmetry of CD units, leading to the destruction of intramolecular H-bonds and an increase in the population of free secondary OH groups of CD units; $I_{4}$ decreases as a result of the diminished population of primary OH groups of CD [10]. These results obtained for dry CDNS confirm the regioselective nature of the polymerization process, which favors the cross-linking of primary OH groups of CD units (Figure 2a).

The response to the thermal motion of different $\nu_{OH}$ sub-bands shows the progressive destruction of highly coordinated H-bond networks of interstitial water molecules (decreases in $I_{1}$ and $I_{3}$) and an increase in primary and secondary OH group populations (increases in $I_{2}$ and $I_{4}$). The $I_{4}$ increase is a result of the destructive effect of temperature on the H-bonds involving carbonyl groups of cross-linkers, resulting in an increased population of free primary OH groups which, together with the destructive effect on intramolecular H-bond networks, promotes an increase in $I_{2}$. The population of intracavity water molecules is the most isolated one; thus, the $I_{5}$ value remains practically unchanged, regardless of any external stimuli [27]

**Figure 4:**
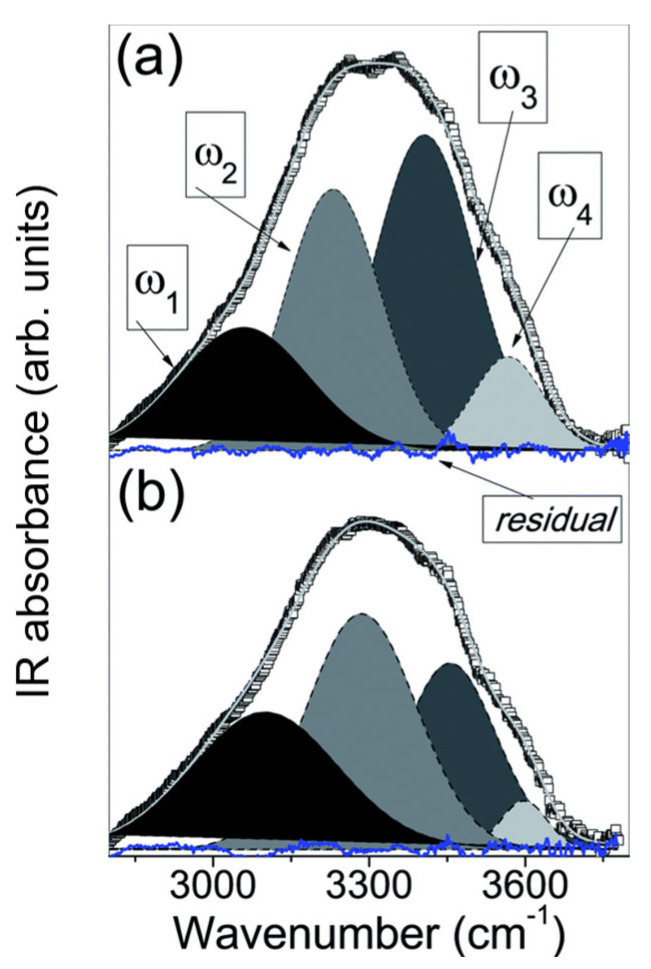
Results of $\nu_{OH}$ decomposition in β-CD:EDTA (1:6) hydrogels at (**a**) h = 4.3 and (**b**) h = 20.2. Adapted from [32], licensed under CC BY 3.0.

$\nu_{OH}$ decomposition in CDNS hydrogels presents a different set of sub-bands: $\omega_{1}$ of symmetric $\nu_{OH}$ ($\nu_{OH(sym)}$), $\omega_{2}$ of asymmetric $\nu_{OH}$ ($\nu_{OH(asym)}$), $\omega_{3}$ of non-in-phase stretching of tetrahedral structures (“bifurcated” H-bonds, where one hydrogen atom is involved in the formation of two H-bonds) and $\omega_{4}$ of water molecules involved in partially broken H-bond networks or not involved in the formation of coordinated networks (Figure 4). $\omega_{1}$ and $\omega_{2}$ together create a bulk-like water molecule population, creating tetrahedral H-bond networks, whereas $\omega_{3}$ and $\omega_{4}$ can be labeled as a non-bulk-like water molecule population that is involved in the formation of less coordinated H-bond networks [32,40,41].

**Figure 5:**
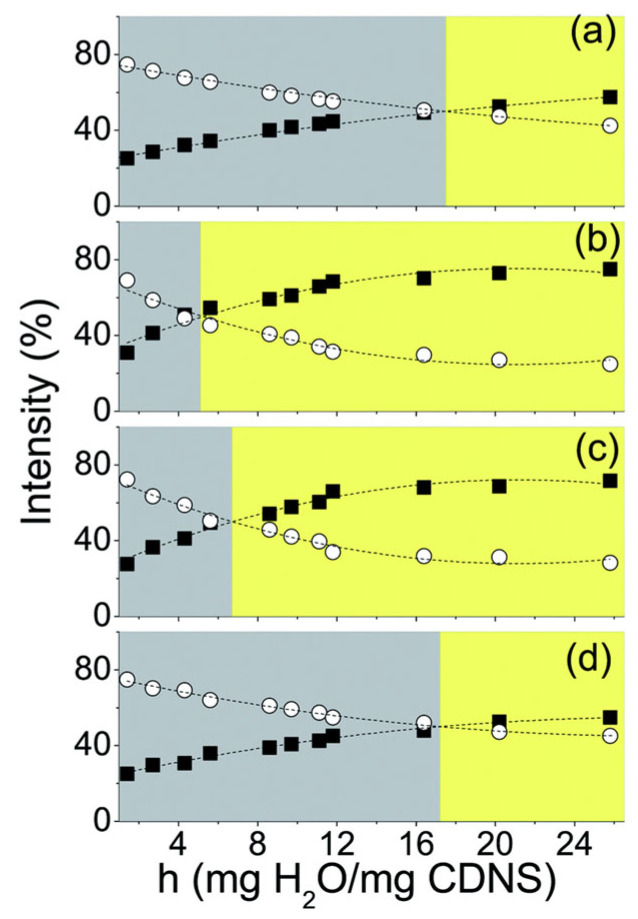
Evolution of bulk-like ($I_{1}$ and $I_{2}$) and non-bulk-like ($I_{3}$ and $I_{4}$) populations of (**a**) β-CD:EDTA (1:4), (**b**) β-CD:EDTA (1:6), (**c**) β-CD:EDTA (1:8), and (**d**) β-CD:EDTA (1:10) with increasing h. CDNSs exist in a hydrogel form (gray part) and undergo transition into liquid form after surpassing $h_{cross}$ (green part). Clear 1:*n* dependence can be seen, with the lowest $h_{cross}$ value observed at a 6-fold excess of EDTA. Adapted from [32], licensed under CC BY 3.0.

For CDNS hydrogels with increasing h, bulk-like water populations ($I_{1}$ and $I_{2}$) increase at the expense of non-bulk-like water molecule populations ($I_{3}$ and $I_{4}$). This creates a cross-over point ($h_{cross}$), where bulk-like H-bond networks become favored over non-bulk-like ones [34,35,41]. For $h<h_{cross}$, water binds inside the pores and at the surface of the CDNS, which results in swelling and hydrogel formation. At $h_{cross}$, water binding sites are saturated and the CDNS cannot absorb more water, which, at $h>h_{cross}$, results in a gel–sol phase transformation to a liquid suspension [34,41]. Thus, $h_{cross}$ can be treated as a descriptor of the maximum hydration level for which the CDNS remains in hydrogel form, beyond which a gel–sol phase transition occurs. $h_{cross}$ values obtained for different CDNS types show that at a 4-fold excess of cross-linker, β-CD:PMDA becomes saturated at lower h ($h_{cross}\approx 4.7$) compared to β-CD:EDTA ($h_{cross}=17$), and thus, EDTA-based CDNSs present better water absorption and swelling properties [32,34]. $h_{cross}$ of β-CD:EDTA hydrogels is the lowest at 6-fold excess of EDTA, where water absorption capacity is the poorest, which is related to the highest reticulation and the poorest pore-enlarging ability, confirming that the macroscopic properties of β-CD:EDTA hydrogels (water absorption ability, hydrogel rigidity) strongly depend on 1:*n* (Figure 5). [32,35,41]. $h_{cross}$ values of α-CD:EDTA and γ-CD:EDTA are smaller than those of β-CD:EDTA, indicating that β-CD creates networks with better water absorption properties compared to its differently sized homologues. Additionally, $h_{cross}$ of γ-CD:EDTA shows 1:*n*-independent behavior, suggesting that the gel–sol phase transition becomes independent of the cross-linking degree for bigger macrocycles [35].

Populations of bulk-like and non-bulk-like water molecules reach an equilibrium state, with the saturation effect occurring in all populations at high h. The same saturation effect is observed as all sub-bands upshift and reach a plateau with increasing h, which is connected to the increasing pore size of CDNSs up to a maximum point, where they are no longer able to enlarge despite a continuous increase in water content [32,34,35,41].

$\nu_{OH}$ band decomposition allows the probing of the behavior of different water molecule populations with increasing temperature. $I_{1}$ and $I_{2}$ decrease and $I_{3}$ increases at elevated temperatures, implying the distortion of well-structured H-bond networks due to thermal motion. $I_{4}$ increases and reaches a plateau, suggesting that the water molecules involved in partially broken H-bond networks are involved in binding at the CDNS surface [28]. Selective probing of this population can be performed by hydrating CDNSs using deuterated water, which leads to the creation of DHO molecules, which emerge due to isotopic exchanges between D_2_O and superficial hydrogen atoms of CDNSs. The DHO band appears between 2700 and 3700 cm^−1^, whose line differs from the standard $\nu_{OH}$, with behavior remaining the same. The DHO mode upshifts with an intensity decrease at higher temperatures. The same behavior is observed for DHO-mode sub-bands ($I_{1}+I_{2}$ reduction, $I_{3}+I_{4}$ increase) suggesting an increase in the non-bulk-like water molecule population at the expense of coordinated bulk-like networks due to the destructive effect of temperature [38,40]. The evolution of percentage intensities with temperature leads to a cross-over temperature $T_{cross}$ for which the non-bulk-like water population becomes favored over the bulk-like population, similar to $h_{cross}$. For CDNS hydrogels hydrated with water, $T_{cross}$ ≈ ~340 K, whereas for β-CD:PMDA and β-CD:EDTA hydrogels hydrated with deuterated water, $T_{cross}$ ≈ ~280 K and ≈ ~300–310 K, respectively. This result suggests the existence of a H-bond network within the CDNS network even at very low temperatures. $T_{cross}$ of the β-CD:PMDA (1:4) hydrogel hydrated with Na_2_CO_3_ solution in D_2_O is higher compared to that of the hydrogel hydrated with deuterated water (~280 K vs. ~307 K). This implies that pH influences the destructive effect of temperature, with more stable H-bonds at increased pH, which is a result of an increased mesh size favoring the swelling process in a basic environment [38,39,40].

### 2.2. $\nu_{C=O}$—Carbonyl Stretching Band

#### 2.2.1. General Analysis

Quantitative analysis of the $\nu_{C=O}$ band related to cross-linker carbonyl groups requires the preparation of spectral data. For qualitative analysis, spectra have to be normalized to the intensities of $\nu_{C-O}$ (~1030 cm^−1^, FT-IR) and $\nu_{{CH}_{2}}$ (~2917 cm^−1^, Raman), which are chosen as internal standards since the bonds related to those modes are not involved in the polymerization process [10,27,29,30,33]. In the spectral region of $\nu_{C=O}$, no CD-unit-related signals appear; however, $\delta_{HOH}$, appearing at ~1640 cm^−1^, overlaps with $\nu_{C=O}$. This can be avoided by hydration with D_2_O, whose bending mode $\delta_{DOD}$ appears at ~1210 cm^−1^ and enables separate examination of the effect of water confinement on $\nu_{C=O}$ of the polymer [30,38,39,40]. Additionally, Raman-active PMDA ring breathing modes $\nu_{(C=C)1}$ and $\nu_{(C=C)2}$ (~1580 cm^−1^ and ~1600 cm^−1^) must be subtracted to avoid any overlapping effects [38,39]. $\delta_{HOH}$, $\nu_{(C=C)1}$ and $\nu_{(C=C)2}$ carry valuable structural information, which is described in depth later.

The evolution of $\nu_{C=O}$ of CDNS hydrogels with increasing 1:*n* consists of an initial downshift, with the minimum obtained for a 6-fold excess of cross-linker, suggesting that a H-bond network involving carbonyl bonds is created at the expense of the bulk-like H-bond network. A further 1:*n* increase results in a $\nu_{C=O}$ upshift related to the branching of CD units and enhanced steric hindrances, leading to the disruption of the coordinated H-bond network of carbonyl bonds at the expense of the reorganization of water molecules into interconnected H-bond networks [30,38].

As temperature or h increases, $\nu_{C=O}$ of β-CD:PMDA hydrogels upshifts with an intensity increase. This is a result of increased thermal motion inducing a destructive effect on the H-bond network involving carbonyl groups of the cross-linker [30,39]. These results are consistent with $\nu_{OH}$ evolution observed for β-CD:PMDA hydrogels [28,38].

#### 2.2.2. Sub-Band Analysis

$\nu_{C=O}$ decomposition reveals four contributions: $\omega_{CO1}$ (~1700 cm^−1^, IR-active) and $\omega_{CO2}$ (~1705 cm^−1^, Raman-active), related to ester groups, and $\omega_{CO3}$ (~1705 cm^−1^, IR-active) and $\omega_{CO4}$ (~1730 cm^−1^, Raman-active), assigned to carboxyl groups [33]. These modes are the same for dry and hydrated CDNSs. Differences in sub-band positions are due to a combination of the different principles of the used methods, different properties of CDNSs and different functions used during the fitting procedure—Voigt functions are predominately used for IR data, and Lorentzian functions for Raman data (Appendix A.1) [10,29].

Similarly to $\nu_{OH}$ sub-bands, areas under the $\nu_{C=O}$ sub-bands ($I_{CO1}-I_{CO4}$) reflect the population of carbonyl species. The total estimated intensities ($I_{CO1}+I_{CO3}$ for IR, $I_{CO2}+I_{CO4}$ for Raman) increase with 1:*n*, with the maximum occurring at a 6-fold excess of cross-linker, confirming the highest reticulation for that molar ratio [27,29]. Relative intensities ($I_{CO1}/I_{CO3}$ for IR, $I_{CO2}/I_{CO4}$ for Raman) behave in the same manner, confirming that the excess of ester bonds with respect to free carboxyl groups is most pronounced at a 6-fold excess of cross-linker, at which point polymerization is saturated [27,29]. This behavior is presumably due to increasing steric effects hindering further reticulation (and formation of new ester linkages) after surpassing a 6-fold excess of cross-linker [29,30].

### 2.3. $\delta_{HOH}$—Water Bending Mode

$\delta_{HOH}$ (~1640 cm^−1^) is related to water molecules not involved in the formation of coordinated H-bond networks [38,40,41]. $\delta_{HOH}$ is more intense in IR, is well reproduced using Voigt functions [39] and behaves inversely to $\nu_{OH}$: a downshift represents an increase and an upshift represents a decrease in the size of the less interconnected water molecule population; thus, $\delta_{HOH}$ can be used as an indicator of coordinated water molecule network formation [28,40,43]. $\delta_{HOH}$ of β-CD:PMDA hydrogels downshifts with an intensity increase with 1:*n*, with the minimum at a 6-fold excess of PMDA, which is consistent with the previously observed destructive effect of cross-linking on the H-bond network. A further increase in 1:*n* results in an upshift and intensity reduction, which suggests reconstruction of the H-bond network due to the favored formation of water–water interactions [28,39]. On the other hand, $\delta_{HOH}$ of β-CD:EDTA hydrogels undergoes a maximum upshift at a 6-fold excess of EDTA, suggesting the formation of highly coordinated H-bonds, which might be related to the higher water absorption ability of β-CD:EDTA compared to β-CD:PMDA, which overcomes the destructive reticulation effect observed for β-CD:PMDA [40].

$\delta_{HOH}$ upshifts and shows an intensity reduction with increasing h of EDTA-based hydrogels, which is consistent with the evolution of $\nu_{OH}$ with h [32,41]. The opposite trend (downshift with intensity increase) is observed with increasing temperature for β-CD:PMDA hydrogels, showing that the reorganization of water molecules into less coordinated networks is favored with increasing thermal motions [38].

$\delta_{HOH}$ linearly downshifts with increasing temperature at different rates depending on 1:*n*. The steepest slope is observed for β-CD:PMDA (1:6), indicating the biggest population of non-bulk-like oriented water molecules. The polymerization process is most efficient with a 6-fold excess of PMDA due to a suitable proportion of OH groups of CD units and activated carbonyls of PMDA, which results in the formation of smaller pores and therefore the greatest destructive effect on coordinated H-bond networks. Higher 1:*n* favors the rearrangement of water molecules into bulk-like networks, which reduces the rate at which the downshift decreases [38,40].

The presence of carboxylic groups of PMDA in the CDNS network implies the pH dependence of the water absorption process, which leads to an increase in mesh size and water absorption capacity. The hydration of CDNSs with solutions of Na_2_CO_3_ (10–25% *w*/*w*) increases swelling as a result of electrostatic repulsion of negative charges in the CDNS network due to the deprotonation of carboxylic groups in basic conditions [42]. This is represented by the upshift of $\delta_{HOH}\;of\;$β-CD:PMDA hydrogels with increasing pH, in which, due to mesh size enlargement, the size of the bulk-like water population increases at the expense of the non-bulk-like one. This is the link between microscopic pH-dependent pore enlargement and macroscopic water absorption abilities. In addition, $\delta_{HOH}$ linearly downshifts with increasing temperature independently of pH, showing a similar destructive effect of thermal motion on H-bonds irrespective of pH changes [43].

### 2.4. $\nu_{C=C}$—Aromatic Stretching of PMDA Ring

In standard Raman spectra of PMDA-based CDNSs, $\nu_{\left(C=C\right)1}$ and $\nu_{\left(C=C\right)2}$ modes are of negligible intensity, which impedes their proper examination. These modes are enhanced in UV Raman experiments, which provide higher sensitivity to the vibrational signals of aromatic moieties due to resonance effects (π-π* transitions) [47]. $\nu_{\left(C=C\right)1}$ is much more polarized compared to $\nu_{\left(C=C\right)2}$, which enables the selective probing of the $\nu_{\left(C=C\right)1}$ mode in isotropic Raman spectra and supports the use of $\nu_{\left(C=C\right)2}$ as an internal standard for spectra normalization [39].

The intensities of $\nu_{\left(C=C\right)1}$ and $I_{\left(C=C\right)1}/I_{\left(C=C\right)2}$ reach their maxima at a 6-fold excess of PMDA, at which point the perturbation of C-H aromatic groups of the PMDA ring is presumably the highest. Due to strong repulsive forces, hydrophobic C-H groups regulate—via rigidity and pore-enlarging properties—the water absorption capacity of CDNSs [39]. This shows the importance of the balance between hydrophobic and hydrophilic groups for the water absorption properties of CDNSs.

The intensity of $\nu_{\left(C=C\right)1}$ is enhanced with h, suggesting that aromatic C-H groups of PMDA play a significant role in the formation of non-conventional (C-H⋯O-H) H-bonds due to increased hydrogen donor ability, which makes $\nu_{\left(C=C\right)1}$ a descriptor of the perturbation of vibrational dynamics of aromatic C-H groups of PMDA induced by water molecules [39,43]. With increasing h, the $I_{\left(C=C\right)1}/I_{\left(C=C\right)2}$ ratio rises to h=8, where a plateau is obtained. This result suggests that the perturbation of the vibrational dynamics of C-H groups by water molecules is substantial at lower h and vanishes above h=8, probably due to the formation of bulk-like H-bond networks [39]. These results suggest that the perturbation of hydrophobic C-H groups is extended over a larger number of hydration shells compared to carbonyl hydrophilic groups [38]. On the other hand, the intensities of $\nu_{C=O(ester)}$ and $\nu_{C=O(carbox)}$ of β-CD:PMDA hydrogels hydrated with Na_2_CO_3_ solution are independent of h, in contrast to hydrogels hydrated with water, which implies that solvation effects are dominated by interactions involving hydrophobic parts of CDNSs with solvent molecules rather than hydrophilic parts of the network [43].

A downshift with an intensity decrease in $\nu_{\left(C=C\right)1}$ is observed with increasing temperature, implying strengthening of H-bonds in which aromatic C-H groups of PMDA are involved. This result is counterintuitive; however, the disruption of highly coordinated H-bond networks due to increased thermal motion leads to an increase in the non-bulk-like water molecule population, which reinforces the perturbation experienced by aromatic CH groups of PMDA, leading to the formation of non-conventional H-bonds [42].

A significant downshift of $\nu_{\left(C=C\right)1}$ is observed as the pH of the β-CD:PMDA hydrogel increases, which confirms the strong involvement of aromatic C-H groups of PMDA in the formation of non-conventional H-bonds and suggests the involvement of pH in H-bond network dynamics [42,47]. The pH dependence of $\nu_{\left(C=C\right)1}$ is more evident at higher temperatures and suggests that the force constant of the ring breathing mode tends to decrease more rapidly with temperature at higher pH, which in turn suggests that at basic pH, the gel–sol phase transition is promoted through the activation of C-H bonds of PMDA units towards solvation, which makes CDNSs temperature-sensitive [47].

A summary of the structural evaluation of the four most significant vibrational modes is provided in Table 2.

### 2.5. UV Raman in H-Bond Dynamics

The 1500–1800 cm^−1^ range is enriched with spectral data carrying information about changing molecular dynamics under environmental or intrinsic stimuli. Every vibrational mode has a well-defined phase that is lost over time due to collisions with other modes within the system. This process is governed by the dephasing time $\tau_{deph}$, whose reciprocal represents the collision rate of the solvent molecules on the vibrating chemical groups [39]. $\tau_{deph}$ of $\nu_{\left(C=C\right)1}$ is a parameter that provides detailed insight into the dynamics of aromatic C-H groups of the PMDA ring and can be extracted from UV Raman spectra using Kubo–Anderson functions (Appendix A.2) [39,42].

With increasing h, $\tau_{deph}$ of $\nu_{\left(C=C\right)1}$ of the β-CD:PMDA (1:4) hydrogel increases to $h^{*}\approx 5$, above which it becomes hydration-independent. $h^{*}$ corresponds to the abovementioned $h_{cross}$ [34]—this suggests that the collision rate reaches a maximum value due to saturation of the water binding sites of the CDNS at $h^{*}\approx h_{cross}$, with the formation of a highly coordinated H-bond network at $h>h_{cross}$, which results in a gel–sol phase transition [39]. The same conclusion can be drawn based on the evolution of ${(I}_{C=O(ester)}+I_{C=O(carbox)})/I_{\left(C=C\right)2}$ with the h, which increases to $h^{*}$ and reaches a plateau, meaning that the perturbation of carbonyl groups becomes hydration-independent after saturation of the water binding sites of the CDNS and the gel–sol phase transition [39].

$I_{\left(C=C\right)1}/I_{\left(C=C\right)2}$ increases with temperature at T > 335 K, where C-H groups are more exposed to collisions. On the other hand, ${(I}_{CO(ester)}+I_{CO(carbox)})/I_{\left(C=C\right)2}$ is temperature-independent, which suggests that hydrophobic groups of CDNS are more sensitive to H-bond rearrangement than hydrophilic ones. $\tau_{deph}$ of $\nu_{\left(C=C\right)1}$ becomes temperature-dependent at $T^{*}$ ≈ 337 K, above which a decrease in $\tau_{deph}$ is observed, showing an increased collision rate at higher temperatures, consistent with the increased perturbation of CH groups at higher temperatures. This implies that at a specific temperature $T^{*}$, the hydrogels are thermally activated, which increases the accessibility of water molecules to hydrophobic groups of the CDNS [47].

The $\tau_{deph}$ of $\nu_{\left(C=C\right)1}$ β-CD:PMDA (1:4) hydrogels hydrated with Na_2_CO_3_ solution decreases with temperature, contrary to β-CD:PMDA (1:4) hydrogels hydrated with water. This result implies a reduction in the hydrophobicity of aromatic C-H groups of PMDA at basic pH [43]. The $T^{*}$ value decreases with increasing pH, which is consistent with the increased hydrogen donor ability of C-H groups, whereas the $\tau_{deph}$ decrease at elevated temperatures is pH-independent, showing its dependence only on thermal motions [42].

Drug loading affects the structural arrangement of the CDNS system, which results in changes in vibrational data. $\nu_{\left(C=C\right)1}$ and $\nu_{C=O}$ of caffeine-loaded β-CD:PMDA (Caf@β-CD:PMDA) hydrogels downshift with an intensity reduction with increasing temperature. The temperature dependence of $\nu_{C=O}$, not occurring for non-loaded CDNSs, is a result of non-covalent interactions between the CDNS and drug [44]. The shift of $\nu_{C=O}$ is more pronounced compared to that of $\nu_{\left(C=C\right)1}$, suggesting that in the presence of the drug (especially hydrophilic Caf), the hydrophilic groups of PMDA display enhanced sensitivity to the rearrangement of water–polymer interactions compared to the hydrophobic groups, which are responsive to changes in h and pH. In the case of Caf@β-CD:PMDA hydrogels, $\tau_{deph}$ of $\nu_{\left(C=C\right)1}$ is temperature-independent up to the activation temperature $T^{*}$, above which it linearly decreases. This behavior is similar to non-loaded CDNSs [42], but their $T^{*}$ values differ—~337 K for non-loaded CDNSs and ~330 K for loaded CDNSs—showing the effect of thermo-responsive behavior on the establishment of non-covalent drug–polymer interactions. The rate at which $\tau_{deph}$ of $\nu_{\left(C=C\right)1}$ decreases at elevated temperatures is the same for non-loaded and loaded CDNSs, which therefore present the same thermo-responsive behavior, only activating at different temperatures. Drug loading overcomes the pH dependence of $T^{*}$, showing that the drug–polymer interactions are dominant over the mesh size changes occurring as a result of a pH increase [44].

### 2.6. Comparison of FT-IR and Raman in CDNS Studies 

It is hard to directly compare the results of IR and Raman measurements due to similarities of the two methods. However, each of these two techniques can be treated as more preferred for examination of different vibrational modes. That information and its consequences is summarized in Table 3.

## 3. Other Scattering Methods

### 3.1. Introduction to Scattering

Scattering events are represented by momentum transfer Q [56]:

(1) $$Q=\frac{4\pi \sin\theta }{\lambda }$$

**Figure 6:**
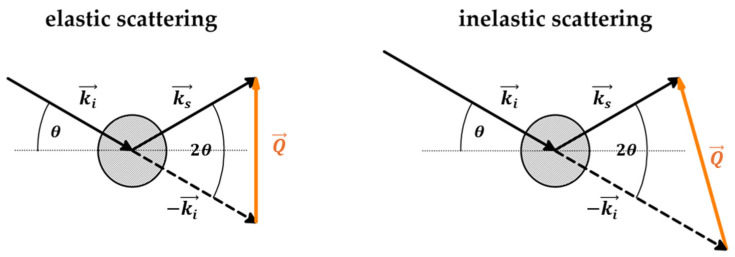
Elastic and inelastic scattering triangles. During elastic scattering, the incident $k_{i}$ and scattered $k_{s}$ eigenvectors are of the same energy ($E_{i}=E_{s}$); thus, scattering occurs without energy exchange. During inelastic scattering, energy transfer leads to scattering with energy exchange ($E_{i}\neq E_{s}$).

The name *momentum transfer* is used interchangeably with *scattering vector*, as it represents the wavevector (eigenvector) of scattering. The scattering vector describes the geometry of the scattering event and depends on the wavelength of incident radiation and the scattering angle [57]. Depending on the energy exchange during the event, scattering can be elastic, occurring without energy transfer (ΔE=0), or inelastic, occurring with energy transfer (ΔE≠0) (Figure 6) [58,59].

Elastic scattering is used mostly to examine molecular structure, whereas inelastic scattering is used to evaluate molecular dynamics. Scattering types can also be divided based on the relationships of scattered waves. Scattering is coherent when scattered waves are in-phase (i.e., maintain the same waveform over time), which leads to wave interference and an increase in signal intensity. Incoherent scattering originates from non-simultaneous scattering over scattering centers, which leads to wave decay [59]. Coherent scattering provides information about the relative positions of atoms and is used to examine crystal structures and density correlations. Incoherent scattering can be used to probe the movement of individual atoms over time and evaluate single-atom dynamics. Applications of different scattering types are presented in Table 4.

Taking into account the magnitude of the scattering angle 2θ (Figure 6), momentum transfer is denoted by q (lowercase q) for small 2θ and Q (capital q) for large 2θ. Large momentum transfer (Q) is related to short wavelengths (or large scattering angles); thus, scattering experiments are able to probe small-scale local structures. On the other hand, small momentum transfer (q) is related to long wavelengths (or small scattering angles), which are able to probe large-scale structures, such as the structural features of a material. Although Raman scattering operates at very low q, it probes high-energy excitations, and, thus, it is able to resolve the vibrational modes of bonds or chemical groups. Table 5 presents the characteristics of different scattering methods [60,61].

### 3.2. Low-Frequency Raman Spectroscopy

#### 3.2.1. Boson Peak Evolution

**Figure 7:**
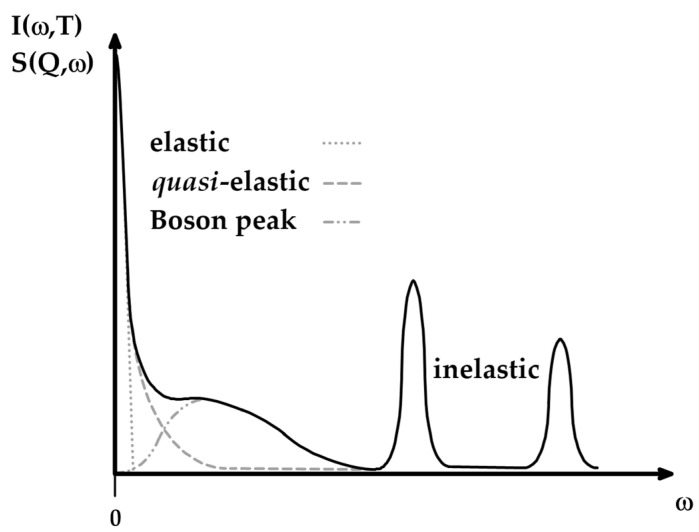
Shape of Raman/INS intensity with detailed description of each spectral contribution.

Disorder-induced scattering is a phenomenon that occurs when particles scatter due to imperfections in a material. In disordered systems, all vibrational modes contribute to the scattering process; this is in contrast to crystalline systems, where only chosen modes are active due to crystal momentum selection rules. This theory is applied in the analysis of the Raman spectrum in the low-frequency region $\left(\tilde{\nu }=0-200\;{cm}^{-1}\right)$, where the contribution of different spectral components to the shape of the spectrum is evident (Figure 7). At $\tilde{\nu }=0\;{cm}^{-1}\;(\omega =0)$, the spectrum is dominated by the elastic contribution (ħω=0) and is represented by a sharp signal of high intensity, almost non-visible on the spectrum. The elastic signal decays rapidly with increasing $\tilde{\nu }\;(\omega )$, where broadening of the spectrum points towards a quasi-elastic (QE) contribution (ħω≈0), which also decays with increasing $\tilde{\nu }\;(\omega )$, but more slowly than the elastic line. At larger $\tilde{\nu }\;(\omega )$, inelastic contributions (ħω≠0) dominate in the form of sharp signals of different intensities [62]. Notwithstanding this, in disordered systems, Raman intensity in the low-frequency region is a superimposition of QE and inelastic lines [63]:

(2) $$I_{Raman}\left(\omega ,T\right)\propto I_{QE}\left(\omega ,T\right)+I_{Inel}\left(\omega ,T\right)$$

This superimposition hides the inelastic signal in the form of a broad bump called the Boson peak (BP), with a maximum $\omega_{BP}$ in the range 15–30 cm^−1^, representing the collective vibrational dynamics of disordered systems and being related to the elastic properties of the system on the mesoscopic length scale (Figure 6) [25,26]. The QE line is related to the diffusion motions and local disorientations, and its intensity is smaller than that of the Boson peak line. Both spectral contributions are relatively separated within the low-wavenumber region, so the QE contribution can be subtracted from the spectra to extract the data regarding the shape and position of the BP, the upshift of which indicates increasing stiffness of the polymer [31]. Using experimental data and curve-fitting procedures, the QE line can be well reproduced via a Lorentzian centered at the zero wavenumber, and the inelastic line via log-normal distribution functions (Equation (3)) [29,31,64]:

(3) $$I_{Raman}\left(\omega \right)=\frac{A\Gamma }{\Gamma^{2}+\omega^{2}}+Be^{-\left\{\frac{{\left[\ln\left(\frac{\omega }{\omega_{BP}}\right)\right]}^{2}}{2W^{2}}\right\}}+Ce^{-\left\{\frac{{\left[\ln\left(\frac{\omega }{\omega_{vib}}\right)\right]}^{2}}{2D^{2}}\right\}}$$

where A, B and C are the amplitudes of the functions; Γ, W and D are widths (FWHMs) of the Lorentzian and both log-normal functions, respectively; and $\omega_{vib}$ is the inelastic vibrational mode maximum, which is used to resolve possible additional inelastic contributions. Log-normal functions are chosen over the standard Gaussian functions because they provide a more quantitative estimation of $\omega_{BP}$ [31]. Fitting experimental data to Equation (3) shows that the QE contribution does not significantly affect the shape of the spectral line; thus, the subtraction of the QE line helps in better describing the BP [26,31].

Raman spectral lines in the low-frequency region of CDNSs vary with 1:*n*, which reflects changes in the low-energy vibrational dynamics related to the cross-linking density [25]. $\omega_{BP}$ can be used as a descriptor of the elastic properties related to the vibrational dynamics of different types of CDNSs. For β-CD:PMDA, α-CD:EDTA and β-CD:EDTA, an upshift of $\omega_{BP}$ is observed, with a maximum appearing with a 6-fold excess of cross-linker, with a further downshift at higher 1:*n* [26,29,31,41]. This outcome is consistent with FT-IR and Raman data [27]. The balance between the free OH groups of CD units and the activated carbonyl groups of the cross-linker at a 6-fold excess of PMDA/EDTA results in the formation of CDNSs with the highest stiffness [31]. A further increase in the cross-linker amount results in the branching of CD units and steric hindrances, preventing further growth of the polymeric network and resulting in a decrease in stiffness [26,29,41]. For γ-CD:EDTA, $\omega_{BP}$ is independent of 1:*n*, showing that for larger macrocycles, the cross-linking degree does not influence the elastic properties of the system. This is consistent with the $h_{cross}$ dependence on 1:*n* observed for EDTA-based CDNSs [41].

#### 3.2.2. Vibrational Density of States (VDOS)

The evolution of atomic displacement during vibrations can be described by the displacement–displacement correlation function C(t), measuring the correlation between atomic displacements over time. The Fourier transform of this function is proportional to the vibrational density of states (VDOS, $g\left(\omega \right)$), which is a function that carries information about the distribution of vibrational modes over various frequencies [65]:

(4) $$g\left(\omega \right)\propto \int_{-\infty }^{\infty }C\left(t\right)e^{-i\omega t}dt$$

with i being imaginary number.

Peaks of the $g\left(\omega \right)$ function represent the concentration of vibrational modes within the system. The inelastic contribution to Raman intensity $I_{Inel}\left(\omega ,T\right)$ in the low-frequency region is related to VDOS via the relation derived by Shuker and Gammon [66,67]:

(5) $$I_{Inel}\left(\omega ,T\right)\propto \frac{g^{R}\left(\omega \right)[n\left(\omega ,T\right)+1]}{\omega }$$

(6) $$n\left(\omega ,T\right)=\frac{1}{e^{\left(-\frac{\hbar \omega }{k_{B}T}\right)}-1}$$

where ħ is the reduced Planck constant (ħ=h/2π, $h=6.626\times 10^{-34}\;J/s$), and $k_{B}$ is the Boltzmann constant ($k_{B}=1.380\times 10^{-23}\;J/K$).

The term $n\left(\omega ,T\right)$ is the Bose–Einstein occupation number, often referred to as the Bose temperature factor or Bose population factor, which, in this case, describes the population of vibrational modes excited at frequency ω at temperature T [68]. The term $g^{R}\left(\omega \right)$ is a convolution of the VDOS function g(ω) and the frequency-dependent light-to-excitation coupling factor C(ω), also called the Raman coupling factor [68,69]. On the basis of the Shuker–Gammon model, the convolution can be simplified to [66]

(7) $$g^{R}\left(\omega \right)=C\left(\omega \right)g(\omega )$$

where C(ω) is a weighting function, showing how strongly each mode contributes to Raman intensity. Its value is unknown, but it can be evaluated experimentally through simultaneous measurements in inelastic neutron scattering experiments (mentioned below). To rule out the temperature dependence of Raman data, conversion to reduced Raman intensity $I^{red}\left(\omega \right)$ is needed, which also indirectly provides the pure VDOS function [31,67,70]:

(8) $$I^{red}\left(\omega \right)=\frac{I_{inel}(\omega ,T)}{[n\left(\omega ,T\right)+1]\omega }\approx \frac{g^{R}\left(\omega \right)}{\omega^{2}}=\frac{C\left(\omega \right)g(\omega )}{\omega^{2}}$$

$I^{red}\left(\omega \right)$ can be modeled using Equation (8) and used to analyze the structural defects, anisotropy and glass-like dynamics of CDNSs via the BP. $\omega_{BP}$, visualized by $I^{red}\left(\omega \right)$ of β-CD:PMDA, upshifts with increasing 1:*n*, which indicates the increased stiffness of CDNSs on a mesoscopic length scale due to the strong connectivity of covalent ester bonds [33]. α-CD:EDTA and β-CD:EDTA spectral data overlap, creating a BP master curve without any adjustable parameters—this shows that the BP depends only on elastic properties and not on the microscopic structure of the material [31,33].

### 3.3. Inelastic Neutron Scattering (INS)

Similarly to Raman measurements, neutron scattering provides information about the structure and dynamics of the system on the picosecond time scale (compared to the femtosecond time scale of Raman scattering). INS intensity is reflected by the double-differential cross-section (Appendix A.3) [56,59,63,71,72,73]:

(9) $$I(Q,\omega )={\left(\frac{d^{2}\sigma }{d\Omega d\omega }\right)}_{tot}=\frac{k_{s}}{k_{i}}\frac{\sigma_{coh}}{4\pi }S_{coh}\left(Q,\omega \right)+\frac{k_{s}}{k_{i}}\frac{\sigma_{inc}}{4\pi }S_{inc}(Q,\omega )$$

Equation (9) shows that scattering intensity depends on coherent and incoherent contributions, each consisting of incident and scattered wavevectors, $\sigma_{coh}$ and $\sigma_{inc}$, and coherent and incoherent dynamic structure factors, $S_{coh}(Q,\omega )$ and $S_{inc}(Q,\omega )$, respectively, each containing information about spatial and temporal fluctuations in particle distribution within the system related to the chosen scattering type.

Since $S_{inc}(Q,\omega )$ encodes atomic movement and vibrations, isolation of the inelastic incoherent signal registered during INS experiments enables the extraction of the VDOS function from the incoherent scattering term (Table 4). $S_{inc}(Q,\omega )$ consists of several terms: the Debye–Waller factor (DWF) $e^{-2W(Q,T)}$; the elastic incoherent structure factor (EISF) $A_{0}\left(Q\right)$, describing the type of motion related to scattering (confined or translational), convoluted with the Dirac delta function $\delta \left(\omega \right)$, ensuring a contribution to elastic scattering of only zero-energy-transfer events (at ω=0); the QE dynamic structure factor $S_{QE}\left(Q,\omega \right)$ convoluted with the EISF term; the inelastic dynamic structure factor $S_{inel}(Q,\omega )$; and the resolution function of the instrument R(Q,ω) convoluted with all other terms [63,71,74,75]:

(10) $$S_{inc}\left(Q,\omega \right)\approx e^{-2W(Q,T)}\left\{A_{0}\left(Q\right)\delta \left(\omega \right)+\left[1-A_{0}\left(Q\right)\right]S_{QE}\left(Q,\omega \right)\;+S_{inel}(Q,\omega )\right\}⨂R(Q,\omega )$$

The Debye–Waller factor (DWF) reveals the reduction in spectral intensity as a result of increased thermal motion at elevated temperatures since it is related to the MSD $\langle u^{2}\rangle$ of the nuclei:

(11) $$2W\left(Q,T\right)=Q^{2}\langle u^{2}\rangle$$

$S_{inc}\left(Q,\omega \right)$ presents characteristics similar to low-frequency Raman spectra (Figure 6). The inelastic term of Equation (10) is proportional to the VDOS function through a similar relation to Raman data (Equation (5)) [63]:

(12) $$S_{Inel}\left(Q,\omega \right)\propto \frac{g\left(\omega \right)[n\left(\omega ,T\right)+1]}{\omega }$$

The difference lies in the Raman coupling factor $C\left(\omega \right)$, which is absent in INS data. Thus, by comparing Raman and INS intensities, $C\left(\omega \right)$ can be calculated according to Equation (7). Raman and INS data provide raw intensities that cannot be directly compared due to the dependence of the excitation intensity on the population of excited modes at a given temperature. Thus, the conversion to a temperature-independent quantity needs to be conducted. The dynamical susceptibility $\chi \left(\omega \right)$ describes how the system responds to an external disturbance as a function of the frequency of the oscillation that causes it. $\chi \left(\omega \right)$ consists of real and imaginary parts:

(13) $$\chi \left(\omega \right)=\chi^{\prime }\left(\omega \right)+i\chi^{\prime \prime }(\omega )$$

The real part $\chi^{\prime }\left(\omega \right)$ describes how much external energy the system can store (elastic response), while the imaginary part $\chi^{\prime \prime }(\omega )$ describes how this energy is absorbed and distributed within the system (inelastic response). The temperature-independent INS intensity obtained using the Bose–Einstein distribution equals

(14) $$\chi_{INS}^{\prime \prime }\left(\omega \right)=\frac{S_{inel}(\omega )}{n\left(\omega ,T\right)+1}\approx \frac{g\left(\omega \right)}{\omega }$$

where $S_{inel}(\omega )$ is a product of the integration of $S_{inel}(Q,\omega )$ over the entire angular range. Conversion isolates the actual intensities strictly related to the dynamic behavior of the system (independent of DWF) and enhances spectral features appearing at low frequencies [33]. The $\chi_{INS}^{\prime \prime }\left(\omega \right)$ curve is similar to that of $I^{red}\left(\omega \right)$; therefore, the BP mode can be observed using INS methods [76].

Merging Equations (10) and (12) provides an approximation of the master formula for the Q-dependent VDOS function [75]:

(15) $$g(Q,\omega )\propto \frac{\omega S_{inel}(Q,\omega )}{Q^{2}[e^{-2W\left(Q,T\right)}]\left[n\left(\omega ,T\right)+1\right]}$$

The integration of the following equation over the entire angular range with fixed Q values enables the calculation of VDOS g(ω).

The relationship between 1:*n* and CDNS rigidity can be extracted from INS—the maxima of $g\left(\omega \right)$ and $\chi_{INS}^{\prime \prime }\left(\omega \right)$ of β-CD:PMDA hydrogels decrease with increasing 1:*n*, suggesting that reticulation (formation of covalent ester bonds and H-bonds) hinders the vibrational dynamics of the CDNS. This indicates that the functional properties of CDNSs may be tunable and suggests that the mesoscopic and macroscopic properties of hydrogels are interconnected [33]. INS data of β-CD:PMDA collapse into a single master curve irrespective of 1:*n*, confirming the previous conclusion from Raman experiments that the BP relies on elastic properties, not on the microscopic structure of the system, which confirms the high reliability of both methods in studying the vibrational dynamics of CDNSs [33].

### 3.4. Brillouin Light Scattering (BLS)

BLS refers to the inelastic scattering of incident photons with quasi-particles—phonons—governing the collective motion of a material, revealing mass oscillation (acoustic) modes in a solid or liquid state. BLS and Raman use photons of similar wavelengths, but the frequency shifts enable the distinction of signals. BLS frequency shifts are in the GHz range, which makes them sensitive to low-energy excitations associated with various atomic chain oscillation types, while Raman frequency shifts are in the THz range, corresponding to the high-energy excitations of oscillations corresponding to specific chemical bonds [77,78,79]. BLS experiments provide information about the elasticity and local viscosity through the analysis of the Brillouin frequency $\omega_{B}$ corresponding to the velocity of collective motion waves. The wavelengths of Brillouin acoustic modes are a few hundred nanometers, while the collective movements propagate over a distance of a few wavelengths; thus, Brillouin scattering experiments can reveal the dynamics of the system at macroscopic lengths. Brillouin wavelengths correspond to small wavenumbers (${\tilde{\nu }}_{Brillouin}=0.1-6\;{cm}^{-1}$), which makes BLS a complementary technique to low-frequency Raman [79].

The phonon wavevector q in BLS can be expressed similarly to that in standard elastic light scattering (Equation (1)); however, since acoustic phonons create periodic variations in the material density, the wavevector relies on the refractive index of the sample (n) [80]:

(16) $$q=\frac{4\pi n\sin\theta }{\lambda }$$

The measurements are usually conducted in backscattering geometry (2θ=180°), where the momentum transfer is the highest (sinθ in Equation (16) cancels out). This allows for the probing of phonons of shorter wavelengths, which improves the resolution and sensitivity of the method [81]. Usually, incident and scattered light are of the same polarization, which allows the selective probing of longitudinal phonons (oscillating parallel to wave propagation) related to changes in material density caused by compression or elongation, separately, from transverse phonons (oscillating perpendicular to wave propagation), which are related to shear-like deformations [82]. Scattering events over longitudinal and transverse phonons are well separated in BLS spectra, with the former occurring at higher frequencies with stronger intensity.

In ideal conditions, sound waves propagate without any attenuation mechanisms (undamped acoustic modes), and very sharp maxima are expected at a frequency $f_{L}$ related to the sound propagation velocity $c_{L}$:

(17) $$c_{L}=\frac{2\pi f_{L}}{q}=\frac{\omega_{B}}{q}$$

In real scenarios, the light is scattered by attenuated sound waves representing local density fluctuations caused by phonons, indicating that the material resists compression or extension. It is measured by the longitudinal modulus $M\left(\omega \right)$, describing the behavior of the system in a more complex environment, where the dynamics of the system disturb the propagation of acoustic modes. For materials exhibiting viscoelastic properties, the longitudinal modulus is a sum of real and imaginary parts [80]:

(18) $$M\left(\omega \right)=M^{\prime }\left(\omega \right)+iM^{\prime \prime }(\omega )$$

$M\left(\omega \right)$ is a concept similar to dynamical susceptibility $\chi \left(\omega \right)$. The real part $M^{\prime }\left(\omega \right)$ carries information about the reversible elastic response (stiffness), while the imaginary part $M^{\prime \prime }(\omega )$ describes the viscous response (viscosity) of the system, which is irreversible due to the loss of energy as heat (damping). Therefore, high $M^{\prime }\left(\omega \right)$ is related to high stiffness, while high $M^{\prime \prime }(\omega )$ indicates high viscosity. Both parts of the longitudinal modulus are related to the specific parameters of the Brillouin peak—the frequency at the maximum $\omega_{B}$ and linewidth $\Gamma_{L}$ (Brillouin line shape is of Lorentzian type) [80,81,83,84]:

(19) $$M^{\prime }\left(\omega \right)=\rho {c_{L}}^{2}=\rho \frac{{\omega_{B}}^{2}}{q^{2}}$$

(20) $$M^{\prime \prime }\left(\omega \right)=\rho c_{L}\Gamma_{L}=\frac{\rho \omega_{B}\Gamma_{L}}{q}$$

with ρ being the mass density of the system.

The behavior of attenuated sound waves leads to signal broadening, which can be described by the fluctuation–dissipation theorem linking the response of the system to external disturbance (dissipation) with the spontaneous fluctuations of the system in thermal equilibrium. The intensity of Brillouin spectra can be modeled as follows [84,85]:

(21) $$I_{B}\left(\omega \right)=\frac{I_{0}}{\omega }\frac{M^{\prime \prime }\left(\omega \right)+\omega \eta_{L}}{{\left[\frac{{\rho \omega }^{2}}{q^{2}}-M^{\prime }\left(\omega \right)\right]}^{2}+{\left[M^{\prime \prime }\left(\omega \right)+\omega \eta_{L}\right]}^{2}}$$

with $I^{0}$ being an amplitude factor dependent on the scattering cross-section.

The Brillouin intensity $I_{B}\left(\omega \right)$ represents how strongly a material fluctuates at a frequency ω, while the longitudinal modulus terms with longitudinal viscosity $\eta_{L}$ describe how a material responds to and dissipates energy. The interplay between these parameters determines the position, height and width of the Brillouin peak, which provides the most important information about the viscoelastic properties of the system. Thus, this region of the spectrum must be described with especially high accuracy. The frequencies around the Brillouin peak can be well reproduced by the model of a damped harmonic oscillator (DHO), which oscillates with decreasing amplitude due to energy dissipation (similarly to the attenuated acoustic modes) [81,82,84]:

(22) $$I_{B}\left(\omega \right)=\frac{I_{0}}{\pi }\frac{\Gamma {\omega_{B}}^{2}}{{\left[\omega_{B}^{2}-\omega^{2}\right]}^{2}+{\left[\omega \Gamma \right]}^{2}}⊗R(\omega )$$

Fitting BLS experimental data for EDTA-based hydrogels to Equation (22) resolves two acoustic modes: soft and hard contributions associated with structural changes in solvent-rich and polymer-rich environments, respectively. For PMDA-based hydrogels, a single signal is observed, suggesting that over a length scale of a few hundred nanometers, the EDTA-based CDNS structure presents a more heterogeneous nature compared to PMDA-based CDNSs [47,48].

$\omega_{B}$ of β-CD:PMDA upshifts with increasing 1:*n*, implying a reduction in the polymer’s flexibility at a 6-fold excess of PMDA. These results are consistent with low-frequency Raman data, showing that both $\omega_{BP}$ and $\omega_{B}$ can be used as descriptors of elastic properties—$\omega_{BP}$ over a few nanometers (mesoscopic range) and $\omega_{B}$ over a few hundred nanometers (macroscopic range). Additionally, the positions of $\omega_{BP}$ and $\omega_{B}$ do not significantly change depending on the macrocycle type, suggesting that the stiffness of the polymer does not rely on the macrocycle size in the way that it varies with the type and amount of cross-linker [26].

$\omega_{B}$ of β-CD:PMDA hydrogels upshifts with increasing pH, following a course similar to the trend for different 1:*n*, suggesting the formation of a more rigid, interconnected network as a result of shifting from acidic to basic pH. On the other hand, $\omega_{B}$ downshifts with increasing temperature, indicating decreasing stiffness of the CDNS [47]. The slope of the $\omega_{B}$ decrease, being a descriptor of the softening rate of a material upon heating, is higher at more basic pH, showing that CDNS solvation and the gel–sol phase transition are favored at higher pH. BLS results are consistent with Raman data regarding the $\nu_{\left(C=C\right)1}$ evolution with temperature, indicating a correlation between the molecular and collective properties of hydrogels during the gel–sol phase transition. Heating the gel has two effects that lead to the phase transformation—improvement of water accessibility to the hydrophobic CDNS sites (increase in pore size) and rearrangement of intermolecular interactions, leading to the separation of CDNS domains (decreased sound velocity), both leading to enhanced solvation of the system triggered by an increase in pH [47].

Γ is directly related to the relaxation process via the lifetime of H-bonds (Equation (A5)) and thus to the H-bond rearrangement process, which is intensified at higher temperatures. Activation energies of relaxation processes obtained from BLS spectra registered at increasing temperatures using Arrhenius plots show pH independence, which implies that the thermo-responsive behavior of PMDA-based CDNSs is dominated by the solvation of hydrophobic aromatic C-H groups of PMDA rather than reorganization of H-bond networks involving hydrophilic carbonyl groups of the cross-linker [47].

### 3.5. Small-Angle Neutron Scattering (SANS)

SANS experiments are based on small scattering angles (0.1–10°), which enable the probing of materials’ structural inhomogeneities on large length scales (100 nm and more) [86,87]. Scattering occurs due to variations in scattering lengths b arising from differences in nuclear composition within the probe (Appendix A.3). The scattering length is used to calculate the scattering length density ρ (SLD), which is a more general parameter describing the scattering properties of a system:

(23) $$\rho =\sum_{i}n_{i}b_{i}$$

$n_{i}$ is the number of nuclei per unit volume, characterized by different scattering lengths. The difference in SLDs between two regions of material or between two different materials shows how strongly neutrons scatter at interfaces or boundaries (or between molecules and the solvent). This difference is measured by scattering contrast Δρ [88]:

(24) $$\Delta \rho =\rho_{1}-\rho_{2}$$

The larger the contrast, the more intense the scattering, whereas for Δρ=0, the scattering signal vanishes [89]. A large contrast caused by hydrogen atoms can be adjusted by mixing water with D_2_O or using selective deuteration, which can highlight specific structures of the system [90]. Since SANS scattering is elastic, scattering intensity is expressed via the differential coherent scattering cross-section, with Δρ being one of the contributions [56]:

(25) $$I\left(q\right)\propto \frac{{d\sigma }_{coh}}{d\Omega }\left(q\right)={\left|\Delta \rho \right|}^{2}V^{2}P\left(q\right)S\left(q\right)+bkg$$

V is the particle’s volume, $P\left(q\right)$ is a form factor related to the shape and size of individual scatterers (describes how a single object scatters neutrons), $S\left(q\right)$ is a structure factor related to the relative positions and interactions between scatterers, and bkg is background noise. Adjusting the scattering contrast between the scatterers and the solute helps to maximize the scattering intensity through maximization of ${\left|\Delta \rho \right|}^{2}$.

An important feature of SANS experiments is the selective measurement of scattering signals. Usually, the neutron beam is collimated, and thus, coherent scattering is primarily measured. Incoherent scattering is mostly due to hydrogen atoms possessing large $\sigma_{inc}$ (~80 barns) and producing unwanted background noise, which can be minimized by diluting the sample or partially or fully replacing the probe’s protons H 1 for deuterium H 2, whose $\sigma_{inc}$ is practically negligible [56]. Then, the coherent signal can be separated from the incoherent contribution, coming mostly from the signal of the solvent.

SANS experiments reveal structural fluctuations and inhomogeneities on a length scale of hundreds of nanometers. If the system presents two different types of inhomogeneities, $I\left(q\right)$ can be modeled using Lorentzian-type functions via a modified two-correlation length model [91,92,93]:

(26) $$I\left(q\right)=\frac{A}{{\left(1+Z^{2}q^{2}\right)}^{2}}+\frac{B}{1+{\left(\zeta q\right)}^{2}}+bkg$$

A and B are fitting parameters, and bkg is the background contribution to $I\left(q\right)$. The first term represents the long-range behavior of the system according to the Debye–Bueche regime and describes the scattering arising from the spatial inhomogeneities of size Z, appearing in the spectra in the form of an upturn at low q values. Large fluctuations of Z values are observed at low h, with convergence to a constant value of ~350 Å with increasing water content, which can be interpreted as the characteristic length of structural inhomogeneities in CDNS hydrogels [36].

The second term is related to the short-range behavior of the system in accordance with the Ornstein–Zernike regime describing the scattering caused by concentration fluctuations in the network due to the inhomogeneous distribution of chain lengths and cross-linking sites, which is strongly related to polymer–solvent interactions and represented by a broad shoulder in the medium-q range [36,48]. The characteristic length scale $d=\frac{2\pi }{q}$ resembles the distance between scattering centers (cross-links, pores); i.e., repeating structural features occurring within the system. A shift towards lower q in the medium-q-range shoulder is associated with increasing d and enlargement of the polymeric network, which is independent of the macrocycle type and 1:*n* with increasing h, implying a similar enlarging of the CDNS hydrogel structure in response to increasing water content. The course of the curves changes with the macrocycle type and 1:*n* (α-/β-/γ-CD:PMDA and α-/β-/γ-CD:EDTA), showing that the local structure of CDNS hydrogels depends on the chemical composition of the CDNS and the water content of the hydrogel [36,48].

The ζ value corresponds to the correlation length ζ in the short range, which can be considered an estimate of the mesh size of the polymer network. ζ increases with h and reaches a plateau for both PMDA- and EDTA-based CDNSs; however, the ζ values at the plateau differ, i.e., ~10–30 Å for PMDA-based CDNSs and ~40–50 Å for EDTA-based CDNSs, because EDTA can produce more flexible polymers with larger pores compared to PMDA. This suggests that the choice of cross-linker enables the modulation of CDNS pore size. This is consistent with the higher $h_{cross}$ values observed for EDTA-based CDNSs compared to PMDA-based ones, confirming the higher water absorption capacity of the former. Additionally, the h dependence of ζ varies for different 1:*n*, which confirms that CDNS structural properties can be easily tuned by controlling the synthesis parameters [46]. The ζ value of β-CD:EDTA hydrogels increases rapidly between pH 7 and 8, after which a plateau is obtained—this is related to ionization of the carboxyl groups of EDTA, which creates repulsive forces that enlarge the polymer structure. The plateau suggests that a basic environment promotes pore enlargement irrespective of the specific pH. In the case of PMDA-based CDNSs, the ζ value does not show clear pH dependence, which further confirms the superior water absorption properties of EDTA-based CDNSs [48].

ζ(h) data can be fitted using a sigmoid-like function to extract characteristic structure-related parameters of CDNSs [94]:

(27) $$\zeta \left(h\right)=\frac{C\times {\left(Kh\right)}^{m}}{1+{\left(Kh\right)}^{m}}$$

with m being an adjustable exponent parameter.

The parameter C is related to the maximum volume accessible to the solvent, whose value represents the average maximum inter-chain distance of the polymeric network. The high C value of β-CD:EDTA compared to those of α-/γ-CD:EDTA and β-CD:PMDA indicates the formation of a network with a bigger mesh size. This result shows that the ζ value depends on the macrocycle type: the reduced number of binding sites of the smaller α-CD ring and the offset of high conformational flexibility and a large ring size with a higher number of reactive OH groups of γ-CD result in lower ζ values compared to those obtained for β-CD-based CDNSs.

The parameter K is associated with the swelling rate of the hydrogel. Its values for EDTA-based CDNSs are higher than those for PMDA-based CDNSs, which is consistent with the better water uptake capacity related to greater conformational flexibility of EDTA. At a 6-fold excess of EDTA, the optimal balance between the OH groups of CD units and the carbonyl groups of EDTA creates the densest network, represented by minimum values of parameter C. At an 8-fold excess of EDTA, the branching of CD units results in the formation of dangling EDTA chains and an increase in free carboxylic groups, creating additional water binding sites, which is associated with the maximum value of parameter K. At higher 1:*n*, the K value decreases due to the hindrance of water binding resulting from such excess of cross-linker [46].

BLS and SANS data show that microscopic factors of the internal CDNS structure (macrocycle and cross-linker types, 1:*n*) determine the interactions between CDNS domains responsible for the macroscopic properties of hydrogels [48].

## 4. Nuclear Magnetic Resonance (NMR)

Each nucleus possesses the quantum mechanical property of spin, an intrinsic property of a particle that behaves as if it has angular momentum. A nucleus with a non-zero spin value rotates around a symmetry axis and presents a magnetic dipole moment $\vec{\mu }$ due to the movement of charge q. NMR experiments are based on the excitation of H 1 and C 13 nuclei, which provide the most valuable qualitative information about the structures of organic systems. The application of a fixed magnetic field $B_{0}$ during NMR experiments results in spin reorientation and a split into two spin populations of different energy states, with the low-energy one being more numerous according to the Boltzmann distribution (Appendix A.4) This disproportion is a source of net magnetization $M_{0}$, a resultant of all individual nuclear magnetic moments in the sample. During NMR experiments, oriented magnetic dipole moments are disturbed by the short-term application of a weak external magnetic field at radio frequency (4–900 MHz), called the radio frequency (RF) pulse. Excitation of low-energy spins to high-energy spins occurs only if energy transfer is equal to ΔE, i.e., at a precise resonance angular frequency called the Larmor frequency $\omega_{L}$ [95]:

(28) $$\Delta E=\hbar \omega_{L}=\gamma \hbar B_{0}$$

where γ is the gyromagnetic ratio, which is a link between the magnetic moment and angular momentum considering the charge and mass of the nucleus. Applying 90° (or $\frac{\pi }{2}$) RF pulses excites and flips net magnetization away from the z-axis into the transverse (x-y) plane, which then undergoes precession around the z-axis at the Larmor frequency toward the starting position [96]. Since precession occurs in three-dimensional space, the position of $M_{0}$ can be described in terms of longitudinal magnetization $M_{z}$ (z-axis contribution) and transverse magnetization $M_{xy}$ (x-y plane contribution), each related to a specific relaxation process: $M_{z}$ to spin–lattice relaxation time $T_{1}$ (recovery of the longitudinal magnetization vector towards the thermal equilibrium) and $M_{xy}$ to spin–spin relaxation time $T_{2}-$ (dephasing—vanishing of in-phase nature of transverse magnetization over time) [97]:

(29) $$M_{z}\left(t\right)=M_{0}\times \left(1-e^{-\frac{t}{T_{1}}}\right)$$

(30) $$M_{xy}\left(t\right)=M_{0}\times e^{-\frac{t}{T_{2}}}$$

with $M_{0}$ being equilibrium bulk magnetization.

The reciprocals of both $T_{1}$ and $T_{2}$ produce the spin–lattice relaxation rate $R_{1}$ and the spin–spin relaxation rate $R_{2}$, respectively. Thus, NMR experiments provide insight into the molecular dynamics of the system. The precession of net magnetization of a single nucleus is a component of collective magnetic environmental changes coming from each nucleus in the system. This causes movement of the magnetic field, which generates the electric current being raw NMR data, called free induction decay (FID), which is digitized and Fourier-transformed to create NMR spectra.

### 4.1. Cross-Polarization NMR (CP NMR)

**Figure 8:**
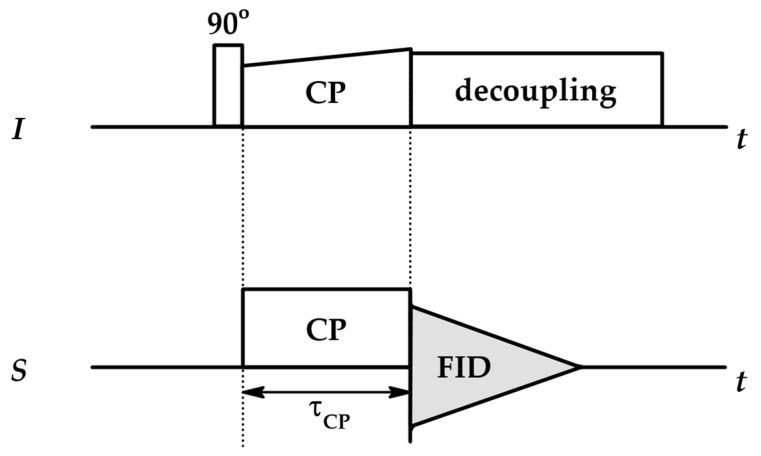
CP pulse sequence divided into high-abundance I spin and low-abundance S spin channels for simultaneous detection and acquisition of data.

NMR-active protons H 1 are the most abundant isotopes of hydrogen (99.98%), which enables registration of H 1 NMR spectra for every organic compound. The abundance of the NMR-active C 13 isotope is ~1.1%, making it hard to register intense C 13 NMR spectra [98]. Cross-polarization NMR (CP NMR) experiments enhance the signal of low-abundance nuclei via magnetization transfer from high-abundance I spins to low-abundance S spins, which is possible due to the existence of homonuclear or heteronuclear dipolar coupling, through-space interactions between the magnetic moments of nuclei. During CP experiments, the probe is placed in a fixed magnetic field $B_{0}$ and excited with a standard 90° RF pulse, after which the spin-locking magnetic field is applied to both low- and high-abundance spins so that the Larmor frequencies of both spins match in the rotating frame. This is the Hartmann–Hahn condition, which must be met to enable magnetization transfer. After the spin-locking pulse is removed, high-abundance spins are decoupled to decrease the interference with the signals of low-abundance nuclei (Figure 8) [97,99].

The enhanced signal intensity of the nucleus is governed by the duration of the spin-locking pulses—contact time $\tau_{CP}$—via the following equation:

(31) $$I(\tau_{CP})=I_{0}(1-e^{-\tau_{CP}/T_{IS}})e^{-\tau_{CP}/T_{1\rho }}$$

with $I_{0}$ being theoretical maximum CP signal intensity (intensity at infinite $\tau_{CP}$).

$T_{IS}$ is the CP time constant characterizing how quickly magnetization is transferred between the spins. It is inversely proportional to the dipolar coupling constant $D_{IS}$; thus, stronger coupling of nearby spins is associated with shorter $T_{IS}$, which produces a high-intensity signal. Fast transfer occurs in rigid structures where dipolar coupling is strong and immobile ($T_{IS}$ of several hundred μs). Dipolar coupling weakens in semi-rigid, disordered systems (e.g., polymers) as mobility increases, leading to longer $T_{IS}$ (up to 1 ms). In liquids molecular motion averages the spin interaction resulting in very long $T_{IS}$ (several ms) which increases even more for long-distance transfers.

$T_{1\rho }$ is the spin–lattice relaxation time in the rotating frame, a relaxation time similar to $T_{1}$, associated with the relaxation of spin-locked spins along the transverse plane. It is used to probe local and slow motions: it is sensitive to motions present in the kHz range (dipolar coupling range), while $T_{1}$ is more sensitive to global molecular changes occurring in the MHz range. The decay of the signal during CP NMR experiments is governed primarily by $T_{1\rho }$ (Equation (31)).

By conducting a series of NMR experiments with various $\tau_{CP}$, the evolution of $T_{IS}$ and $T_{1\rho }$ can be traced. The efficiency of magnetization transfer depends on internuclear distances, conformational changes and rotational dynamics; therefore, variable contact time (VCT) CP NMR experiments provide data based on which we can determine the molecular dynamics of the system, e.g., interactions between CDNSs and an incorporated drug. To extract this information, the experimental curves are fitted to the classical I−S or $I-I^{*}-S$ model (Appendix A.5) [99].

^1^H-^13^C VCT CP MAS NMR data of β-CD:EDTA hydrogels show that $\tau_{CP}$ values are small (ranging from 100 to 1100 μs), suggesting a fast cross-polarization characteristic for rigid structures. CP kinetics for non-loaded CDNSs follow the classical I−S model, with similar $T_{1\rho }$ values of the CD unit and carbonyl group carbons indicating the homogeneous nature of the polymer. The CP kinetics of ibuprofen sodium-loaded β-CD:EDTA (IbuNa@β-CD:EDTA) hydrogels follow the $I-I^{*}-S$ model, with $T_{1\rho }$ values of IbuNa carbons rising with increasing $\tau_{CP}$. The VCT profiles of loaded and free CDNSs fit different dynamic models—changes in dynamics after drug loading suggest that the drug and CDNS are in contact and that magnetization transfer occurs due to the strong heteronuclear dipolar interactions in the rigid system between the protons of the drug and the carbons of the CDNS, indicating the formation of a supramolecular structure [45].

### 4.2. Diffusion NMR

**Figure 9:**
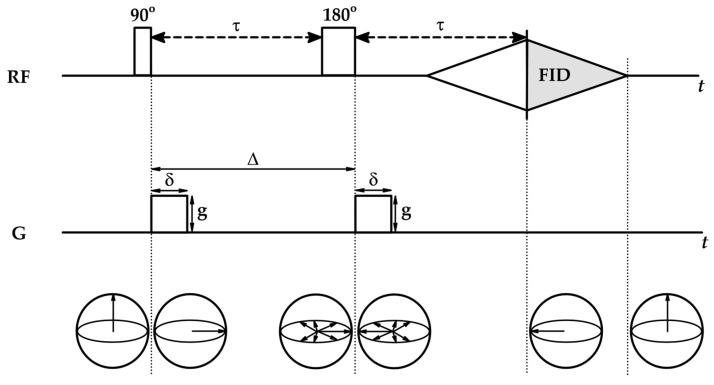
PGSE pulse sequence divided into RF pulse and magnetic field gradient channels, with visualized changes of magnetization transfer during the sequence.

The application of $B_{0}$ orients all spins into a singular plane and makes dipolar coupling observable. Then, the RF pulse orients spins such that the dipolar coupling can be registered by the spectrometer. In a homogeneous system, spins excited by a 90° pulse show the same Larmor frequency. In reality, structural differences within the probe lead to local variations in the magnetic field, leading to a variety of precession rates ($\omega_{L}$) and a faster dephasing process. The transverse relaxation time $T_{2}^{*}$ describes this dependence:

(32) $$\frac{1}{T_{2}^{*}}=\frac{1}{T_{2}}+\frac{1}{T_{inhom}}=\frac{1}{T_{2}}+\gamma \Delta B_{0}$$

where $T_{inhom}$ is the decay caused by magnetic field inhomogeneities, which, in combination with fast dephasing, leads to the loss of FID. To avoid this, pulsed field gradient spin echo (PGSE) experiments are used (Figure 9). They start with the application of a constant magnetic field $B_{0}$, followed by a 90° RF pulse and the dephasing of spins at different rates. At time τ after the first pulse, a 180° pulse (or π pulse) is applied, which flips the spins around the x-axis or y-axis, resulting in the reversal of spins—fast spins, which were in front of slow spins after the 180° pulse, try to “catch” them. Spins preserve their frequencies; thus, at the time τ after the second pulse, the spins rephase, creating a spin echo signal [96]. In PGSE experiments, the primary FID is not registered—the new FID is the spin echo signal, independent of the field homogeneities, and decays with $T_{2}$, not $T_{2}^{*}$ [99].

After each RF pulse, magnetic field gradient pulses are applied: the first one is used to dephase the net magnetization, while the second one is applied to rephase it. Gradient pulses used in PGSE experiments are usually the same and are characterized by the amplitude g, duration δ and time between them Δ (Figure 9). When the positions of spins change due to diffusion during time Δ, the rephasing gradient is not able to fully rephase the net magnetization, which results in signal attenuation, reflected via the Stejskal–Tanner equation [95,96]:

(33) $$I=I_{0}e^{-\gamma^{2}g^{2}\delta^{2}Dt_{eff}}$$

with $I_{0}$ being the theoretical maximum signal intensity.

Particles undergo Brownian motions, and their displacement can be characterized by diffusion time Δ. However, the spins experience different gradient pulses as they move, meaning that the magnetic field gradient does not dephase spins uniformly. The correction of diffusion time is provided via effective diffusion time (or observation time) $t_{eff}$:

(34) $$t_{eff}=\Delta -\frac{\delta }{3}$$

According to the Stejskal–Tanner equation (Equation (33)), the intensity of the NMR signal is a function of observation time $t_{eff}$ and instrumental NMR parameters: gradient pulse amplitude g and duration δ. Therefore,

(35) $$I\left(q,t_{eff}\right)=I(0,t_{eff})e^{-q^{2}Dt_{eff}}$$

where $q^{2}=\gamma^{2}g^{2}\delta^{2}$. The diffusion coefficient provides insight into the dynamics of the particles due to its direct relationship with MSD [95]:

(36) MSD=2nDt

where n is the number of spatial dimensions. In NMR, we consider only the movement of the spins along the z-axis. Thus,

(37) $$MSD=2Dt=\langle z^{2}(t_{eff})\rangle$$

$\langle z^{2}(t_{eff})\rangle$ is the average squared distance traveled by the molecule during $t_{eff}$, meaning that the evolution of MSD can be tracked by a series of experiments using different $t_{eff}$ values.

In isotropic systems where Fickian motion occurs, MSD increases linearly with time; however, in systems where the classic diffusion regime fails, MSD depends on alternative motion regimes via the relation

(38) $$MSD=2D^{\prime }t^{\alpha }$$

where $D^{\prime }$ is the generalized diffusion coefficient. The value of the diffusion exponent α indicates the type of diffusion regime occurring in the system: for 0<α<1, diffusion is shifted towards the anomalous subdiffusive regime, where particles move more slowly than in standard diffusion. For α=1, diffusion is isotropic and unrestricted and undergoes Fickian laws of motion, while for α>1, the anomalous superdiffusive regime dominates, where particles move faster than in standard diffusion.

Combination of Equations (35) and (37) provides the relationship between the intensity of the NMR signal and the MSD of the spins:

(39) $$I\left(q,t_{eff}\right)=I(0,t_{eff})e^{-\frac{q^{2}\langle z^{2}(t_{eff})\rangle }{2}}$$

For experimental purposes, the relation is transformed into

(40) $$\ln\frac{I(q,t_{eff})}{I(0,t_{eff})}=-\frac{1}{2}q^{2}\langle z^{2}(t_{eff})\rangle$$

MSD values calculated through linear regression of Equation (40) are used to calculate diffusion exponents α via a log–log plot of Equation (38) [36].

**Figure 10:**
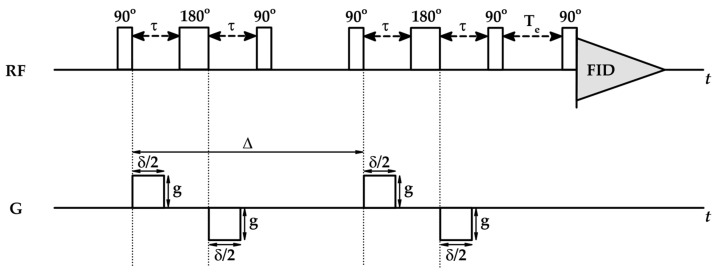
BPP-LED pulse sequence divided into RF pulse and magnetic field gradient channels.

Quick switching on and off of the strong magnetic field gradient pulses leads to disturbance of $B_{0}$, which induces the electric currents within the probe known as eddy currents, which, on their own, create small magnetic fields further increasing the heterogeneity of $B_{0}$. Eddy currents do not decay instantly but persist for milliseconds after gradient pulses, which strongly influences the rephasing process and is a significant confounder to a clean NMR signal. To get rid of magnetic inhomogeneities, bipolar pulse longitudinal eddy current delay (BPP-LED) sequences are used consisting of two 90°-180°-90° RF pulses (Figure 10) [100,101].

90° RF pulse flips the magnetization into transverse plane, after which a positive magnetic field gradient dephases the spins. Similarly to PGSE experiments, 180° RF pulse reverses the spins, after which negative magnetic field gradient refocuses the spins, which cancels out motion-related phase shifts leaving only random diffusion effects. Another 90° RF pulse stores the magnetization in z-axis, while spins are allowed to diffuse. Then, another 90°-180°-90° RF pulse sequence retrieves information about the position of spins after diffusion, after which eddy currents dissipate (LED delay, $T_{e}$) with final 90° RF pulse restoring the magnetization into transverse plane enabling FID registration. Overall, BPP-LED sequence is more specialized than PGSE sequence, which allows to dispose of any artifacts affecting the NMR signal quality. Table 6 summarizes typical experimental parameters of diffusion NMR techniques used in CDNS examination.

Equation (33) is the foundation of diffusion-ordered spectroscopy (DOSY), which can be used to calculate diffusion coefficients of individual groups of atoms within the molecule [102]. It can be used to study water dynamics within the CDNS network—diffusivity data from DOSY experiments conducted on β-CD:PMDA hydrogels confirm the existence of more mobile (higher D) bulk-like water molecules confined within nanochannels and non-bulk-like water molecules bound inside CD cavities or polar binding sites with decreased mobility (smaller D) [25]. MD simulations of β-CD:PMDA in water identified a third type of water molecule whose behavior fits surface water molecules—a D value between bulk-like and non-bulk-like populations—with MSD remaining in a plateau in the initial steps of the simulation and then rapidly increasing, reflecting reentry towards bulk water. Theoretical and experimental D values are in agreement within the order of magnitude, which confirms the reliability of NMR techniques in studying molecular dynamics of CDNSs [103].

Drug molecules in solution exhibit unrestricted diffusion (α=1.00), which changes after their incorporation into the CDNS network. For IbuNa@β-CD:EDTA (1:4), α=0.64, indicating an anomalous subdiffusive regime caused by a densely packed structure, which restricts diffusion of the drug. For IbuNa@β-CD:EDTA (1:8), α=1.08, indicating an anomalous superdiffusive regime of the drug. This counterintuitive outcome is a result of the electrostatic potential generated by carboxyl groups of the cross-linker, which is enhanced with increasing 1:*n*. This potential also affects solute molecules, but the changes in their D values are less pronounced compared to those of probe molecules. Excess EDTA during polymerization leads to the branching of CD units and the formation of free EDTA molecules at polymer ends, confirmed by a certain degree of heterogeneity observed in VCT data of β-CD:EDTA (1:8) [45]. This leads to an increase in the free carboxylic group population and diffusion acceleration. This mechanism explains the enhanced D values of fluorescein observed at higher 1:*n* for β-CD:PMDA hydrogels [25]. These results imply the possible tunability of CDNS drug release properties via 1:*n* and pH due to ionization of carboxyl groups at basic pH [36,37]. In the case of PiroNa@β-CD:PMDA (1:3), α=1.03, suggesting that the drug follows a Fickian diffusion mechanism within the CDNS network. It is hard to compare the diffusion characteristics of different CDNSs based on data obtained for different drugs, which is why a more detailed evaluation is needed using CDNSs synthesized with various types of cross-linkers over the spectrum of 1:*n* and different types of probe drug molecules [50].

Fitting the release data to the Korsmeyer–Peppas kinetic model provides another diffusion parameter, n, which is an indicator of the drug transport mechanism, similar to α [104,105]:

(41) $$\frac{M_{t}}{M_{\infty }}=k_{KP}t^{n}$$

with $k_{KP}$ being the Korsmeyer–Peppas kinetic constant. Depending on the value of n, different diffusion mechanisms might occur (Table 7).

For example, for piroxicam sodium-loaded β-CD:PMDA (1:3) (PiroNa@β-CD:PMDA (1:3)), n=0.296, confirming previously observed Fickian diffusion using NMR methods. The D values of PiroNa@β-CD:PMDA (1:3) obtained from NMR data and release profile studies (using the kinetic model based on conservation laws for hydrogel and solution phases described in Appendix A.6) are of the same order of magnitude, indicating that drug dynamics maintain the same behavior on the microscopic and macroscopic time scales [50]. These results also show that NMR experiments are a reliable method to predict release dynamics and elucidate drug–polymer interaction mechanisms [50].

### 4.3. Fast Field Cycling (FFC) NMR Experiments

In the presence of solid material, water molecules present two types of motion—a water molecule can undergo horizontal diffusion at the surface of the material, or a water molecule re-entering the bulk can be replaced by another molecule binding at the surface of the material. The dynamics of water molecules at the surface of the material contribute to spin–lattice relaxation; therefore, fluctuations in magnetic fields over the system caused by structure inhomogeneity translate to dispersion of longitudinal relaxation times $T_{1}$. Due to surface inhomogeneities, the dynamics of water molecules within porous materials vary—molecules bound within nanopores are more constrained than loosely bound ones. As water molecules remain in pores for longer periods, the longitudinal relaxation rate $R_{1}$ decreases by the same order (also true for transverse relaxation, not considered). Thus, FFC-NMR relaxometry can be used as a valid alternative to the classic methods for porous structure evaluation (Brunauer–Emmett–Teller (BET) theory [106], Barrett–Joyner–Halenda (BJH) theory [107]).

**Figure 11:**
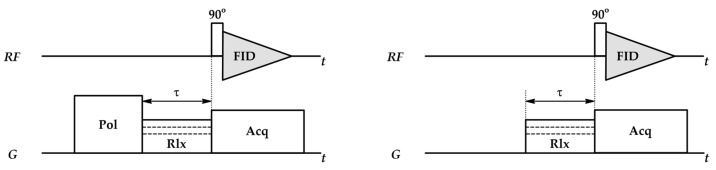
PP (**left**) and NP (**right**) pulse sequences divided into RF pulse and magnetic field gradient channels.

During FFC NMR relaxometry experiments, three consecutive magnetic fields are used: a polarization field (Pol), during which magnetization builds up; a relaxation field (Rlx) of time τ, during which the relaxation process occurs with a new equilibrium of magnetization intensity; and an acquisition field (Acq) applied together with a 90° RF pulse for FID registration (Figure 11). FFC NMR relaxometry experiments modulate the Rlx intensity to measure variations in the $T_{1}$ values of protons over the spectrum of Larmor frequencies. During the pre-polarized (PP) sequence, magnetization is created in stronger Pol, and relaxation occurs in weaker Rlx, where magnetization is strong enough to be detected. In the case of a non-polarized (NP) sequence, no Pol is applied, and magnetization builds up only during Rlx. The magnetization intensities of both sequences can be described accordingly:

(42) $$I_{PP}\left(\tau \right)=I_{0}^{Pol}\times e^{-\frac{\tau }{T_{1}}}$$

(43) $$I_{NP}\left(\tau \right)=I_{0}^{Rlx}\times \left(1-e^{-\frac{\tau }{T_{1}}}\right)$$

with $I_{0}^{Pol}$ and $I_{0}^{Rlx}$ being the theoretical maximum signal intensity emerging from applied Pol and Rlx fields, respectively.

In a situation where $I_{0}^{\mathrm{Rlx}}≳0.5I_{0}^{\mathrm{Pol}}$ , the NP sequence produces magnetization of comparable intensity to the PP sequence, which makes measurements less time-consuming due to a simpler experimental sequence.

$T_{1}$ values extracted from Equation (42) or Equation (43) are used to create a dispersion curve, $R(\omega_{L})$, which shows the relationship between the relaxation rate $R_{1}$ and corresponding Larmor frequency $\omega_{L}$. Dispersion curves can be well reproduced by Lorentzian-type functions [72,99]:

(44) $$R(\omega_{L})=\sum_{i}\frac{c_{i}{\tau_{c}}^{i}}{1+{\left(\omega_{L}{\tau_{c}}^{i}\right)}^{2}}$$

where $\tau_{c}$ is the correlation time, which describes significant changes in the orientation or position of atoms, representing the structural mobility of the system on the microscopic scale. Equation (44) reproduces the dispersion curve of a singular first-order relaxation process. In the case of complex relaxation processes, an additional contribution must be assigned to account for the existence of quadrupolar dips caused by interactions of quadrupolar moments within the system, characteristic of inhomogeneous systems. Using calculated fitting parameters $c_{i}$, the average correlation time of the whole system can be calculated as follows [99]:

(45) $$\tau_{c}=\frac{\sum_{i}c_{i}{\tau_{c}}^{i}}{\sum_{i}c_{i}}$$

Depending on the $\tau_{c}$ value, two contributions are observed for hydrogels hydrated with H_2_O: fast components with longer $\tau_{c}$, related to water molecules tightly bound to the CDNS surface (confirmed by the occurrence of quadrupolar dips in dispersion curves), and slow components with shorter $\tau_{c}$, associated with loosely bound molecules inside CDNS nanochannels. The $\tau_{c}$ values of CDNS hydrogels hydrated with D_2_O are similar to those of dry CDNSs, showing that $\tau_{c}$ carries information regarding the relaxometric behavior of water molecules interacting with CDNSs.

Estimating the pore size of porous materials is difficult due to the unknown pore shape and difficulty in estimating the thickness of the hydration shells of pore walls. To overcome dependence on these parameters, based on the concept of “connectivity” adapted from soil studies [99], the pore connectivity index (PCI) was derived, being directly related to pore diameter. The PCI value shows clear $\omega_{L}$ dependence, which suggests that it can be used as an alternative parameter to provide information about the structural and functional properties of CDNSs related to the mobility of water molecules within the pores. These results show that FFC NMR relaxometry, despite not providing a direct measure of structural parameters, is a more reliable and less time- and material-consuming method of evaluating CDNS structures compared to classical porosimetric methods [49].

## 5. Conclusions

**Figure 12:**
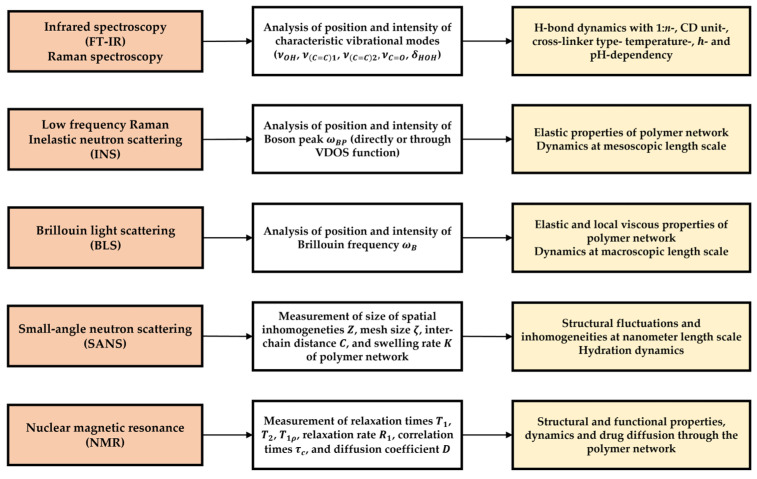
Understanding structural and dynamical behavior of CDNSs via different spectroscopic methods.

The mutual compatibility of different spectroscopic methods makes them great candidates for thorough physicochemical evaluation of CDNS networks. Tracking the evolution of different vibrational modes of polymer and water molecules as a function of 1:*n*, temperature, h and pH shows that FT-IR and Raman spectroscopies provide information about non-covalent water–water and water–polymer interactions arising within polymeric CDNS networks. UV Raman enables easy examination of the vibrational modes occurring within the spectral region of 1500–1800 cm^−1^, which provides insight into the cross-linker’s contribution to H-bond network evolution. The combined use of low-frequency Raman, INS and BLS methods enables examination of the elastic properties of a polymeric network on different length scales. SANS experiments probe the structural inhomogeneities and swelling properties on a microscopic scale, while NMR is the go-to method for understanding the dynamics of CDNS hydrogels and the molecules incorporated within their structures, making it a viable method for examining the diffusion properties of CDNS networks. A summary of the application of spectroscopic methods for examination of CDNSs is provided in Figure 12. Strong links between CDNSs’ microscopic behavior, revealed by spectroscopic methods, and their macroscopic properties, obtained through physical examinations, release profiles and swelling studies, confirm the significantly tunable nature of CDNS properties. This conclusion is of great importance and makes CDNSs great candidates not only for drug transport scaffolds but also for water and soil remediation and toxic waste management, as their properties can be easily adjusted depending on the application.

## Figures and Tables

**Table 1:** Collection of articles constituting the knowledge base of the use of different spectroscopic methods for examining the structure and dynamics of various types of PMDA- and EDTA-based CDNSs. Hydrogel parameters (hydrating solution, hydration level h and pH) are presented in parentheses.

N^o^	CDNS	Form	*1:n* Range	Methods of Examination	Ref.
**1**	β-CD:PMDA	Dry CDNSs and hydrogels (D_2_O)	1:2–1:10	Low- and high-frequency Raman, diffusion NMR (BPP-LED)	[25]
**2**	β-CD:PMDA	Dry CDNSs	1:2–1:10	Low-frequency Raman, BLS	[26]
**3**	β-CD:PMDA	Dry CDNSs	1:2–1:10	ATR-FT-IR, high-frequency Raman	[27]
**4**	β-CD:PMDA	Dry CDNSs and hydrogels (H_2_O, h = 3.3 and 5)	1:4–1:10	ATR-FT-IR, high-frequency Raman	[28]
**5**	β-CD:EDTA	Dry CDNSs	1:4–1:10	ATR-FT-IR, low- and high-frequency Raman	[29]
**6**	β-CD:PMDA	Hydrogels (D_2_O, h = 3.3 and 5)	1:4–1:10	ATR-FT-IR, high-frequency Raman	[30]
**7**	α-/γ-CD:EDTA	Dry CDNSs	1:2–1:10	Low-frequency Raman	[31]
**8**	β-CD:EDTA	Hydrogels (H_2_O, h = 1.4–25.8)	1:4–1:10	ATR-FT-IR	[32]
**9**	β-CD:PMDA	Dry CDNSs	1:2–1:8	Low- and high-frequency Raman, INS	[33]
**10**	β-CD:PMDA	Hydrogels (H_2_O, h = 3–25)	1:4	High-frequency Raman	[34]
**11**	α-CD:EDTA	Hydrogels (H_2_O, h = 2–25.5)	1:2–1:10	ATR-FT-IR	[35]
**12**	IbuNa@β-CD:EDTA	Hydrogels (D_2_O)	1:4, 1:8	Diffusion NMR (PGSE)	[36]
**13**	IbuNa@β-CD:EDTA	Hydrogels (D_2_O)	1:4, 1:8	Diffusion NMR (BPP-LED)	[37]
**14**	β-CD:PMDA	Dry CDNSs and hydrogels (H_2_O and D_2_O, h = 0.4 and 4)	1:4–1:10	ATR-FT-IR, high-frequency UV Raman	[38]
**15**	β-CD:PMDA	Dry CDNSs and hydrogels (H_2_O, h = 2.7–12)	1:4–1:10	ATR-FT-IR, high-frequency UV Raman	[39]
**16**	β-CD:EDTA	Hydrogels (H_2_O and D_2_O, h = 0.4 and 4)	1:4–1:10	ATR-FT-IR, high-frequency UV Raman	[40]
**17**	α-/γ-CD:EDTA	Dry CDNSs and hydrogels (H_2_O, h = 2–25.5)	1:2–1:10	ATR-FT-IR, low-frequency Raman	[41]
**18**	β-CD:PMDA	Hydrogels (H_2_O, D_2_O and 10–25% Na_2_CO_3_ in H_2_O and D_2_O, h = 4, pH = 8.9–10.1)	1:4	ATR-FT-IR, high-frequency UV Raman	[42]
**19**	β-CD:PMDA	Hydrogels (H_2_O, D_2_O and 10–25% Na_2_CO_3_ in H_2_O and D_2_O, h = 4, pH = 8.5–10.1)	1:4	ATR-FT-IR, high-frequency UV Raman	[43]
**20**	Caf@β-CD:PMDA	Hydrogels (15% Na_2_CO_3_ in H_2_O, h = 4, pH = 9.2)	1:4, 1:8	High-frequency UV Raman	[44]
**21**	IbuNa@β-CD:EDTA	Hydrogels (H_2_O)	1:4, 1:8	^1^H-^13^C VCT CP MAS NMR	[45]
**22**	α-/β-/γ-CD:PMDA/EDTA	Hydrogels (D_2_O)	1:2–1:10	SANS	[46]
**23**	β-CD:PMDA	Hydrogels (5–20% Na_2_CO_3_ in H_2_O, h = 4, pH = 6.3–9.8)	1:3	BLS	[47]
**24**	β-CD:PMDA/EDTA	Hydrogels (5–25% Na_2_CO_3_ solutions in H_2_O and D_2_O, h = 1–8, pH = 4.9–9.9)	1:3–1:8	High-frequency UV Raman, SANS, BLS	[48]
**25**	β-CD:HMDI	Dry CDNSs and hydrogels (H_2_O and D_2_O)	1:1–1:4	FFC NMR relaxometry	[49]
**26**	PiroNa@β-CD:PMDA	Hydrogel (10% Na_2_CO_3_)	1:3	Diffusion NMR (BPP-LED)	[50]

**Table 2:** Evolution of vibrational modes depending on chosen intrinsic or external stimuli with description of the associated structural changes.

Vibrational Mode	Structural Changes Related to Evolution of Vibrational Mode	Evolution with Increasing				Ref.
		1:*n*	Temperature	h	pH	
$\nu_{OH}$	Upshift—H-bond lifetime reduction (H-bond weakening), destruction of H-bond network involving OH groups of CD units and water molecules Downshift—H-bond lifetime increase (H-bond strengthening), reorganization of water molecules into coordinated H-bond network	Upshift—maximum at 6-fold excess of cross-linker; downshift	Upshift—maximum at 6-fold excess of cross-linker; downshift	Continuous upshift with intensity decrease	-	[28,32,40,43]
$\nu_{C=O}$	Upshift—destruction of H-bond network involving carbonyl groups of cross-linker Downshift—reorganization of water molecules into coordinated H-bond network involving carbonyl groups of cross-linker	Downshift—minimum at 6-fold excess of cross-linker; upshift	Continuous upshift with intensity increase	Continuous upshift with intensity increase	-	[30,38,39]
$\delta_{HOH}$	Upshift—reorganization of water molecules into coordinated H-bond network Downshift—destruction of H-bond network of water molecules	Downshift with intensity increase—minimum at 6-fold excess of cross-linker; upshift with intensity reduction (PMDA-based CDNS) Upshift—maximum at 6-fold excess of cross-linker; downshift (EDTA-based CDNS)	Downshift with intensity increase (PMDA-based CDNS) Upshift with intensity reduction (EDTA-based CDNS)	Continuous downshift	Continuous upshift	[28,32,38,39,40,41,43]
$\nu_{\left(C=C\right)1}$	Intensity increase—increased perturbation around aromatic C-H groups of PMDA ring induced by water molecules, indicating formation of non-conventional (C-H⋯O-H) H-bonds Intensity decrease—destruction of non-conventional H-bonds involving aromatic C-H groups of PMDA ring	Maximum intensity at 6-fold excess of PMDA	Intensity increase	Continuous downshift with intensity decrease	Continuous downshift	[38,39,42,43]

**Table 3:** Characteristics of FT-IR and Raman methods in terms of sensitivity to different vibrational modes, translating into their possible application in probing structural changes occurring due to drug incorporation within the CDNS network.

Method	FT-IR	Raman
**Probed vibrational modes**	$\nu_{OH}$, $\nu_{C=O}$, $\delta_{HOH}$	$\nu_{\left(C=C\right)1}$, $\nu_{\left(C=C\right)2}$, $\nu_{C=O}$
**Characteristic** **feature**	Sensitive to water presence	Sensitive to double-bonded chemical groups and aromatic moieties
**CDNS type** **preference**	EDTA-based CDNS (stronger water uptake properties)	PMDA-based CDNS (probing non-conventional H-bonds)
**Sensitivity to drug molecule**	Molecules easily forming H-bond with CDNS structure, incorporated within polymer in presence of water molecules	Molecules easily interacting and possibly involved in π-π*

**Table 4:** Scattering types with detailed applications for examining structure and dynamics.

Scattering Type	Carried Information	Probed Systems
elastic coherent	collective atomic positions	macrostructures
elastic incoherent	relative atomic positions	amorphous systems
inelastic coherent	correlated motions	phonons
inelastic incoherent	single-atom motion	diffusion, vibrational density of states (VDOS)

**Table 5:** Comparison of characteristics and applications of different scattering-based methods.

Method	Momentum Transfer Value	Category	Probed Systems
**Raman**	$\sim 10^{-6}\;{Å}^{-1}$	q	optical phonons (local vibrations)
**BLS**	$\sim 10^{-4}\;{Å}^{-1}$	q	acoustic phonons (elastic moduli)
**SANS**	$0.0005-0.5\;{Å}^{-1}$	q	large-scale dynamics
**INS**	$0.1-10\;{Å}^{-1}$	Q	small-scale dynamics

**Table 6:** Experimental parameters of PGSE and BPP-LED experiments used in diffusion NMR studies of PMDA- and EDTA-based CDNS. G/cm is Gauss per centimeter, alternative unit to SI-preffered tesla per meter T/m.

Pulse Sequence	g[G/cm]	δ[ms]	Δ[s]	$t_{eff}[s$]	T[K]	Ref.
PGSE	53	0.5–2.5	0.1	-	300	[36]
BPP-LED	53.5	1.4–3	0.05–0.2	-	305	[25]
	53.5	1.4–3	0.05–0.17	-	305	[37]
	53	1.3–3	-	0.03–0.11	305	[50]

**Table 7:** Characteristics of different drug transport mechanisms based on Korsmeyer–Peppas model.

n	Drug Transport Mechanism	Physical Principle	Dominant Drug Release Model
≤ 0.45	Fickian diffusion	Fick’s laws	Diffusion-controlled release
0.45<n<0.89	Anomalous transport	Fick’s laws, swelling and relaxation of polymeric network	Swelling-controlled release
0.89	Case II transport	Swelling and relaxation of polymeric network, time-independent	
>0.89	Super-case II transport	Relaxation and erosion of polymeric chains	Relaxation-controlled release

**Figure A1:**
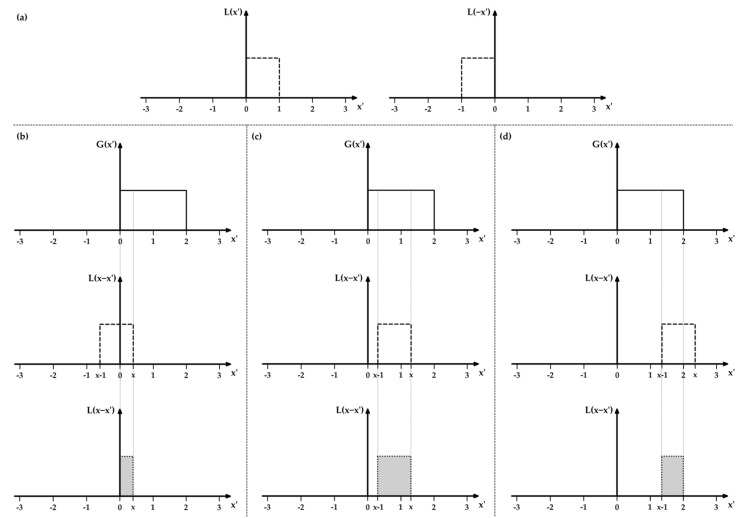
Schematic visual explanation of convolution. (**a**) transformation of Lorentzian line; (**b**–**d**) calculations of Gaussian contributions to the spectral line at chosen x-values.

**Figure A2:**
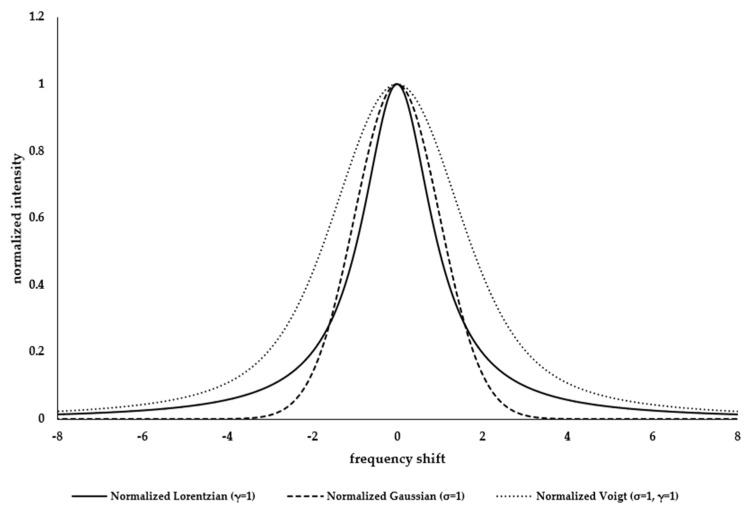
Comparison of shapes of normalized Lorentzian, Gaussian and Voigt functions.

**Figure A3:**
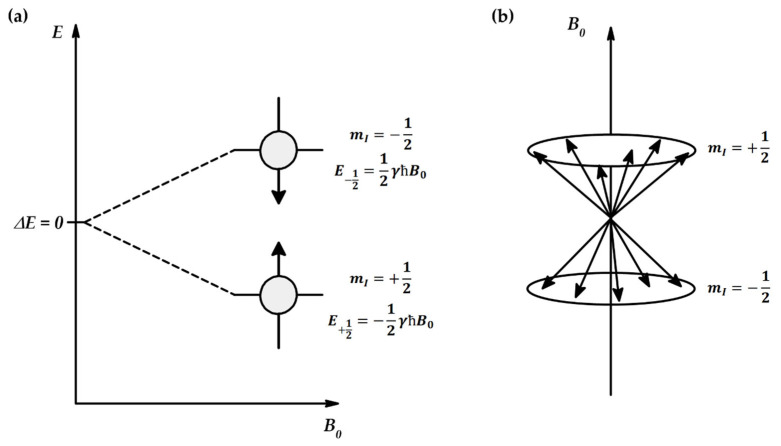
Schematic representation of (**a**) different energy levels for spins with $I=\frac{1}{2}$ and (**b**) their orientation in magnetic field $B_{0}$.

**Figure A4:**
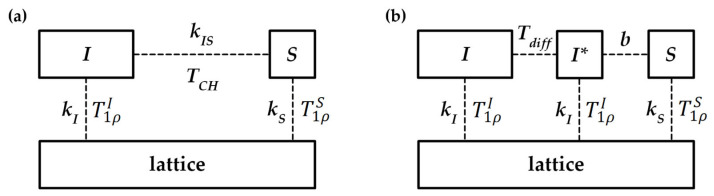
Block diagrams of CP thermodynamics of (**a**) classic I−S model and (**b**) $I-I^{*}-S$ model.

## Abbreviations

The following abbreviations are used in this manuscript:

α-/β-/γ-CD	α-/β-/γ-Cyclodextrin
Acq	Acquisition field
ATR-FT-IR	Attenuated total reflection
BET theory	Brunauer–Emmett–Teller theory
BJH theory	Barrett–Joyner–Halenda theory
BLS	Brillouin light scattering
BP	Boson peak
BPP-LED	Bipolar pulse longitudinal eddy current decay
Caf	Caffeine
CDI	1,1′-Carbonyldiimiazole
CDNS	Cyclodextrin nanosponge
CP	Cross-polarization
DHO	Damped harmonic oscillator
DPC	Diphenyl carbonate
DOSY	Diffusion-ordered spectroscopy
DWF	Debye–Waller factor
EDTA	Ethylenediaminetetraacetic dianhydride
EISF	elastic incoherent structure factor
FID	Free induction decay
FFC	Fast field cycling
FWHM	Full width at half maximum
H-bond	Hydrogen bond
HMDI	Hexamethylene diisocyanate
HR	High resolution
HV	Depolarized
IbuNa	Ibuprofen sodium salt
INS	Inelastic neutron scattering
MAS	Magic angle spinning
MSD	Mean square displacement
NMR	Nuclear magnetic resonance
NP	Non-polarized
PGSE	Pulse gradient spin echo
PiroNa	Piroxicam sodium salt
Pol	Polarization field
PMDA	Pyromellitic dianhydride
PP	Pre-polarized
RF	Radio frequency
Rlx	Relaxation field
QE	Quasi-elastic
SANS	Small-angle neutron scattering
SLD	Scattering length density
TDI	Toluene-2,4-diisocyanate
UV	Ultraviolet
VCT	Variable contact time
VDOS	Vibrational density of states
VV	Polarized

## Appendix A

### Appendix A.1. Resolving Spectral Line Shape

Signals appearing in vibrational spectra are not sharp peaks due to line broadening, which can be modeled using different fitting function types. The two most common functions—Lorentzian and Gaussian—are compared in Table A1.

**Table A1:** Comparison of Lorentzian and Gaussian functions used to describe the broadening of the spectral line.

	Lorentzian Function	Gaussian Function
**Form**	$L\left(x;\gamma \right)=\frac{\gamma }{\pi (x^{2}+\gamma^{2})}$	$G\left(x;\sigma \right)=\frac{1}{\sigma \sqrt{2\pi }}e^{\left(-\frac{x^{2}}{2\sigma^{2}}\right)}$
**Characteristic parameters**	γ	σ
**Relation to signal shape and intensity**	${FWHM}_{L}=\Gamma =2\gamma$	${FWHM}_{G}=2\sqrt{2\ln2}\sigma$

In a situation where the signal presents both Lorentzian and Gaussian contributions to the spectral line, the broadening is described as a convolution of both functions in the form of a Voigt function:

(A1) $$V\left(x;\sigma ,\gamma \right)=\int_{-\infty }^{+\infty }G\left(x^{\prime };\sigma \right)L\left(x-x^{\prime };\gamma \right){dx}^{\prime }$$

where x is a fixed position where the Voigt function is evaluated, while $x^{\prime }$ is an integration variable. The Lorentzian profile $L\left(x^{-}x^{\prime };\gamma \right)$ can be treated as the ideal spectral signal, centered at $x^{\prime }$ and shifted over all values of x, while the Gaussian profile $G\left(x^{\prime };\sigma \right)$ is a weighting function. The inclusion of the variable x enables the sliding of the Lorentzian over the whole Gaussian profile (from −∞ to +∞) and evaluation of how much the Gaussian profile contributes to the spectral signal. Calculations over all x values create the Voigt profile. The convolution concept can be well described visually, as shown in Figure A1, while the shapes of normalized Lorentzian, Gaussian and Voigt profiles (intensity at signal peak equals 1) are compared in Figure A2.

### Appendix A.2. UV Raman Spectroscopy

UV Raman enhances the signal of aromatic modes due to resonance effects. Subtraction of depolarized (HV) Raman intensity from polarized (VV) Raman spectra leads to isotropic Raman intensity $I_{iso}$ composed of an anisotropic contribution to the Raman line arising from asymmetric or distorted vibration modes. $I_{iso}$ is especially useful in studying liquid or amorphous solids, where the molecules are randomly oriented. In the liquid state, the Raman signal principally comes from the intermolecular interactions between the probe and solute molecules. The Kubo–Anderson model approximates the influence of those interactions on the Raman spectral line and enables examination of the system dynamics. $I_{iso}$ can be reproduced as follows [39,42,44]:

(A2) $$I_{iso}\left(\omega \right)=\sum_{i=1}^{N}A_{i}K_{i}(\omega ,\omega_{0}^{i},{\langle \omega^{2}\rangle }^{i},\tau_{c}^{i})+A_{w}W\left(\omega \right)+bkg$$

Equation (A2) is the sum of N Kubo–Andeson functions $K_{i}$, each characterized by the vibrational frequency ω, the unperturbed vibrational frequency of the i-th function $\omega_{0}^{i}$, MSD of the transition wavenumber around the i-th unperturbed frequency ${\langle \omega^{2}\rangle }^{i}$ and the correlation time of the i-th function $\tau_{c}^{i}$. $W\left(\omega \right)$ is the spectral contribution of water (weak in all spectral ranges, can be easily subtracted), $A_{i}$ and $A_{W}$ are scaling factors, and bkg is a background constant. Each Kubo–Anderson function is a variation or convolution of Lorentzian and Gaussian profiles (Appendix A.1.), leading to the master formula [108,109]:

(A3) $$I_{iso}\left(\omega \right)\propto K_{i}\left(\omega ,\omega_{0}^{i},{\left\langle \omega^{2}\right\rangle }^{i},\tau_{c}^{i}\right)\propto \frac{{e^{\left(\alpha_{i}\right)}}^{2}}{\alpha_{i}}\sum_{n=0}^{\infty }\frac{\left({-\alpha }_{i}^{2}\right)}{n!}\frac{\alpha_{i}+\frac{n}{\alpha_{i}}}{{\left(\alpha_{i}+\frac{n}{\alpha_{i}}\right)}^{2}+\frac{{\left(\omega -\omega_{0}^{i}\right)}^{2}}{\left\langle \omega_{i}^{2}\right\rangle }}$$

(A4) $$\alpha_{i}=\tau_{c}^{i}\sqrt{\left\langle \omega_{i}^{2}\right\rangle }$$

with $\left\langle \omega_{i}^{2}\right\rangle$ being the variance of frequency spread around the unperturbed frequency $\omega_{0}^{i}$.

The parameter α in Equation (A4) determines which function type best describes the signal shape: for α≫1, the Gaussian line is predominant, whereas for α≪1, Lorentzian functions are dominant. The correlation time $\tau_{c}$ describes significant changes in the orientation or position of atoms, representing the structural mobility of the system on the microscopic scale. Considering systems with existing H-bonds, α is the spread of the signal’s peak at the central frequency due to changes in H-bond geometry, while $\tau_{c}$ is H-bond lifetime. $\tau_{c}$ is related to the dephasing time $\tau_{deph}$ through the following relation [108,109]:

(A5) $$\tau_{deph}=\frac{1}{\tau_{c}\langle \omega^{2}\rangle }≌\frac{1}{\pi \Gamma }$$

Γ is the Lorentzian linewidth. The reciprocal of $\tau_{deph}$ represents the collision rate of the molecules on the vibrating groups.

### Appendix A.3. Scattering Cross-Sections

Each chemical element is characterized by a scattering length b, creating a theoretical sphere around a particle whose cross-section through the center is recognized as the scattering cross-section σ of barn units (1 barn = $10^{-28}$ m^2^ = 100 fm^2^). σ is the area at which the scattering of the incident particle is most likely to occur; thus, it also describes the probability that a scattering event occurs [56,110]. It strongly depends on the energy of the particle–particle interaction and the type of scattering center. In the simplest scenario, all nuclei present the same b, and scattered waves are in-phase, resulting in coherent scattering characterized by the coherent cross-section $\sigma_{coh}$. In a situation where two or more nuclei coexist within the system with different b, scattering waves are mainly out-of-phase, resulting in incoherent scattering with an incoherent cross-section $\sigma_{inc}$. The greater the difference between b values, the larger the $\sigma_{inc}$. The total scattering cross-section $\sigma_{tot}$ is the sum of coherent and incoherent cross-sections [59,110]:

(A6) $$\sigma_{tot}=\sigma_{coh}+\sigma_{inc}$$

The differential cross-section shows how the scattering probability varies with respect to a single variable. It is the number of particles scattered per second at a particular angle Ω. Measurements over all Ω values provide diffraction patterns carrying structural information of the probe. The double-differential cross-section provides information on two variables—it shows the likelihood of a particle being scattered at a particular angle with specific energy (frequency) and provides insight into the dynamics of the system. Similarly to $\sigma_{tot}$, the total differential and double-differential cross-sections are the sum of coherent and incoherent contributions [58,59]:

(A7) $${\left(\frac{d\sigma }{d\Omega }\right)}_{tot}={\left(\frac{d\sigma }{d\Omega }\right)}_{coh}+{\left(\frac{d\sigma }{d\Omega }\right)}_{inc}$$

(A8) $${\left(\frac{d^{2}\sigma }{d\Omega dE}\right)}_{tot}={\left(\frac{d^{2}\sigma }{d\Omega dE}\right)}_{coh}+{\left(\frac{d^{2}\sigma }{d\Omega dE}\right)}_{inc}$$

### Appendix A.4. Importance of Energy Distribution of Molecules in NMR

NMR experiments are based on the excitation of H 1 and C 13 nuclei, which provide the most valuable qualitative information about the structures of organic systems. For these nuclei, $I=\frac{1}{2}$, meaning that these nuclei exist in two energy states: the “spin up” state $\left(m_{I}=+\frac{1}{2}\right)$ with lower energy $\left(E_{+\frac{1}{2}}=-\frac{1}{2}\gamma \hbar B_{0}\right)$ and the “spin down” state $\left(m_{I}=-\frac{1}{2}\right)$ with higher energy $\left(E_{-\frac{1}{2}}=\frac{1}{2}\gamma \hbar B_{0}\right)$ (Figure A3a) [97].

The formation of two populations of nuclei results in an energy difference between them:

(A9) $$\Delta E=E_{-\frac{1}{2}}-E_{+\frac{1}{2}}=\gamma \hbar B_{0}=\hbar \omega_{L}$$

According to the Boltzmann distribution,

(A10) $$\frac{N_{u}}{N_{l}}=e^{-\frac{\Delta E}{kT}}$$

The lower-energy “spin up” nuclei population $N_{l}$ is larger than the higher-energy “spin down” population $N_{u}$. This disproportion is a source of net magnetization $M_{0}$, being a resultant of all individual nuclear magnetic moments in the sample parallel to the z-axis (Figure A3b).

### Appendix A.5. Kinetics of Magnetization Transfer [99]

The classic I−S model consists of a lattice with a huge heat capacity, isolated S spins and coupled I spins behaving as a single spin (Figure A4a). Magnetization transfer between I and S spins is associated with heat transfer. The thermodynamics of this system requires the implementation of several “heat flow” constants: between spins ($k_{IS}$) and between spins and lattices ($k_{I}$ and $k_{S}$, respectively). The spin–spin “heat flow” rate constant is the reciprocal of the CP time constant $T_{IS}$, while the spin–lattice “heat flow” rate constants are reciprocals of the spin–lattice relaxation times in the rotating frame $T_{1\rho }^{I}$ and $T_{1\rho }^{S}$ for both I and S spins, respectively. Considering this model, the signal of S spins follows the equation

(A11) $$I\left(\tau_{CP}\right)=I_{0}\frac{k_{IS}}{\left(k_{IS}+k_{S}\right)-k_{I}}\left[e^{-k_{I}\tau_{CP}}-e^{-\left(k_{IS}+k_{S}\right)\tau_{CP}}\right]$$

with $I_{0}$ being the theoretical maximum CP signal intensity (intensity at infinite $\tau_{CP}$).

Since the heat capacity of S spins is significantly smaller than I spins, $k_{S}$ is negligible ($k_{S}\ll k_{IS}$). Considering that $k_{IS}+k_{S}>k_{I}$:

(A12) $$I\left(\tau_{CP}\right)=I_{0}\frac{k_{IS}}{k_{IS}-k_{I}}\left[e^{-k_{I}\tau_{CP}}-e^{-k_{IS}\tau_{CP}}\right]=I_{0}\left(\frac{1}{1-\frac{T_{IS}}{T_{1\rho }^{I}}}\right)\left[e^{-\frac{\tau_{CP}}{T_{1\rho }^{I}}}-e^{-\frac{\tau_{CP}}{T_{IS}}}\right]$$

This relation shows that the magnetization of the S spins rises with increasing $k_{IS}$ (decreasing $T_{IS}$) and decays with increasing $k_{I}$ (increasing $T_{1\rho }^{I}$). $T_{IS}$ and $T_{1\rho }^{I}$ can be extracted from Equation (A12) by fitting the experimental curves with the bi-exponential formula.

In a situation where not all I spins behave as a single spin, a subset of $I^{*}$ spins situated between I and S spins is added, leading to the $I-I^{*}-S$ model (Figure A4b), in which the signal is dependent on diffusion time $T_{diff}$. Magnetization transfer between $I^{*}$ and S spins occurs in an oscillatory manner, damped by spin diffusion, which means that the spin diffusion rate $\frac{1}{T_{diff}}$ is a valid variable influencing CP kinetics. Signal intensity in this model is described by the equation.

(A13) $$I\left(\tau_{CP}\right)=I\left(\tau_{CP}\right)=I_{0}e^{-k_{I}\tau_{CP}}\left[1-\frac{1}{2}e^{-\frac{\tau_{CP}}{T_{diff}}}-\frac{1}{2}e^{-\frac{3\tau_{CP}}{2T_{diff}}}\cos\left(\frac{b\tau_{CP}}{2}\right)\right]$$

According to the model, the frequency of the oscillation between $I^{*}$ and S spins depends on the heteronuclear dipolar coupling b due to the orientation of the spins in the external magnetic field. For systems with strong heteronuclear coupling $I^{*}-S$ and weak homonuclear coupling $I-I^{*}$, the system can be described via the $I-I^{*}-S$ model. When $I^{*}-S$ coupling is weak and $I-I^{*}$ coupling is strong, the system follows the classic I−S model.

### Appendix A.6. Extraction of Diffusion Coefficient from Release Profile Data

The diffusion coefficient can be calculated using release data based on the conservation laws for the two phases: hydrogel (G) and solution (S) [111,112,113]. For a three-dimensional system, Fick’s second law is as follows:

(A14) $$\frac{\partial C}{\partial t}=D\left(\frac{\partial^{2}C}{\partial x^{2}}+\frac{\partial^{2}C}{\partial y^{2}}+\frac{\partial^{2}C}{\partial z^{2}}\right)$$

Diffusion through the hydrogel structure can be calculated using the model based on the second Fick’s law in the one-dimensional model in cylindrical geometry:

(A15) $$\frac{\partial C_{G}}{\partial t}=\frac{D}{r}\frac{\partial }{\partial r}\left(r\frac{\partial C_{G}}{\partial r}\right)$$

Besides this core equation, the model also consists of the following:

1. Mass transfer at the cylinder surface with respect to the hydrogel and solution, respectively:

(A16) $$V_{G}\frac{\partial \bar{C_{G}}}{\partial t}=-k_{c}S_{exc}(\bar{C_{G}}-\bar{C_{S}})$$

(A17) $$V_{S}\frac{\partial \bar{C_{S}}}{\partial t}=k_{c}S_{exc}(\bar{C_{G}}-\bar{C_{S}})$$

where $V_{G}$ is volume of hydrogel; $\bar{C_{G}}$ and $\bar{C_{S}}$ are mean drug concentrations in hydrogel and solution, respectively; $k_{c}$ is mass transfer coefficient; and $S_{exc}$ is the exchange surface area (interfacial surface, boundary between the hydrogel and solution).

2. Initial conditions for concentration in the hydrogel and solution, respectively:

(A18) $$C_{G}\left(t=0\right)=C_{G,0}=\frac{m_{G,0}}{V_{G}}$$

(A19) $$C_{S}\left(t=0\right)=0$$

where $C_{G,0}$ is initial drug concentration in hydrogel, and $m_{G,0}$ is initial mass of the drug in hydrogel.

3. Boundary conditions for the center of the cylinder (no flux) and at the cylinder surface (flux continuity, equating the diffusive flux inside the cylinder to the convective mass transfer in the solution):

(A20)
$${\left.\frac{\partial C_{G}}{\partial r}\right|}_{r=0}=0$$

(A21)
$$-D{\left.\frac{\partial C_{G}}{\partial r}\right|}_{r=R}=k_{c}(C_{G}-C_{S})$$

$k_{c}$ is a measure of the rate at which a substance moves from one location or phase to another (it quantifies how easily a substance can be moved across a boundary or interface). The value of $k_{c}$ is computed numerically via approximation algorithms. Then, the diffusion coefficient can be computed through the Sherwood number Sh, representing the ratio of the mass transfer rate to the diffusion rate:

(A22) $$Sh=\frac{2rk_{c}}{D}$$

where r is the particle radius.
